# Cohort-specific disability trajectories among older women and men in Europe 2004–2017

**DOI:** 10.1007/s10433-022-00684-4

**Published:** 2022-02-19

**Authors:** Stefan Fors, Stefania Illinca, Janet Jull, Selma Kadi, Susan P Phillips, Ricardo Rodrigues, Afshin Vafaei, Eszter Zolyomi, Johan Rehnberg

**Affiliations:** 1 Aging Research Center, Karolinska Institutet and Stockholm University, Tomtebodavägen 18A, 171 65 Solna, Stockholm, Sweden; 2 Centre for Epidemiology and Community Medicine, Region Stockholm, Stockholm, Sweden; 3 European Center for Social Welfare Policy and Research, Vienna, Austria; 4 School of Rehabilitation Therapy, Queen’s University, Kingston, ON Canada; 5 Department of Family Medicine, Queen’s University, Kingston, ON Canada

**Keywords:** Disabilities, Ageing, Birth cohorts, Europe

## Abstract

As the population of Europe grows older, one crucial issue is how the incidence and prevalence of disabilities are developing over time in the older population. In this study, we compare cohort-specific disability trajectories in old age across subsequent birth cohorts in Europe, during the period 2004–2017.We used data from seven waves of data from the Survey of Health, Ageing and Retirement in Europe (SHARE). Mixed effects logistic regression models were used to model trajectories of accumulation of ADL limitations for subsequent birth cohorts of older women and men in different European regions. The results showed that there were sex differences in ADL and IADL limitations in all regions for most cohorts. Women reported more limitations than men, particularly in Eastern and Southern rather than Northern and Western Europe. Among men in Eastern, Northern and Western Europe, later born cohorts reported more disabilities than did earlier born birth cohorts at the same ages. Similar patterns were observed for women in Northern and Western Europe. In contrast, the risk of disabilities was lower in later born cohorts than in earlier born birth cohorts among women in Eastern Europe. Overall, results from this study suggest that disability trajectories in different cohorts of men and women were by and large similar across Europe. The trajectories varied more depending on sex, age and region than depending on cohort.

**Supplementary Information:**

The online version contains supplementary material available at 10.1007/s10433-022-00684-4.

## Introduction

As the population of Europe grows older, one crucial issue is how the incidence and prevalence of disabilities are developing over time in older populations. Such trends could have profound consequences for the resources demanded to provide adequate levels of formal and informal care in the future.

As living conditions and lifestyles in Europe have changed over the last decades, more recent birth cohorts entering old age will have experienced very different exposures throughout their lives. This cohort replacement offers one potential source of changing patterns of old-age disability. To the extent that each cohort enters old age with different risk factors for or levels of disabilities, this is likely to shape the incidence and prevalence of disabilities in older populations.

The main aim of this study is to track the disability trajectories of consecutive European birth cohorts through old age and analyze whether levels and trajectories of disabilities differ across cohorts. We will also examine whether these cohort-specific disability trajectories differ for women and men.

Recent studies of trends in disabilities in Europe suggest substantial regional heterogeneity in the trends in old-age disabilities. There is evidence of improving independence in activities of daily living (ADL) among older adults in the Nordic countries (Ahrenfeldt et al. 2018; Christensen et al. 2013; Fors and Thorslund 2015; Hossin et al. 2019), while similar trends are not as clear across other European regions (Ahrenfeldt et al. 2018; Chatterji et al. 2015; Verropoulou and Tsimbos 2017).

Besides regional variance, there is strong evidence that the prevalence of disabilities varies by sex among older adults in Europe with women being more likely to have disabilities (Ahrenfeldt et al. 2018; Scheel-Hincke et al. 2020; Schmitz and Lazarevič 2020). However, despite the sex gap in prevalence, a study based on repeated cross-sectional analyses of several European countries did not find differences in trends in disabilities by sex in Europe (Ahrenfeldt et al. 2018).

These studies are all based on trends and inequalities in the prevalence of disability. As the gendered nature of life-course circumstances has changed substantially over time, it is plausible that differential cohort trends could be observed among men and women. The sex gap in life expectancy has been converging over the last decades (Sundberg et al. 2018; Thorslund et al. 2013). Concurrently sex differences in disability-free life expectancies have been narrowing (Sundberg et al. 2016). This convergence in health outcomes could very well reflect an overall minimizing of sex differences in lifestyles and living conditions. If so, we would expect substantial differences in the magnitude of the sex-gap in morbidities, disabilities and mortality across sequential birth cohorts.

Studies on trends in old-age disabilities across birth cohorts have yielded ambiguous results. A Danish study compared the health and function of two cohorts, born 10 years apart, at age 93–95 (Christensen et al. 2013) reported that the cohort born later, on average, had better cognition and fewer disabilities than the earlier cohort. These improvements were of similar magnitude for women and men. Similarly, a regional Swedish study compared the disability rates, at age 75, of two cohorts born 30 years apart. The results showed that the group born more recently had lower prevalence of disability than did the other cohort. This decrease was especially prominent among women (Falk et al. 2014). These findings align with those from another study based on data from Europe and the USA, showing a general trend of decreasing disability among later born cohorts; however, the cohort trend in England was reversed with an unexpected increase in disability rates for the most recent cohorts (born 1956–1958) (Chatterji et al. 2015). Similarly, other studies from the USA, Europe and Hong Kong have also reported increasing rates of disabilities among later born cohorts (Beller and Epping 2021; Lin et al. 2012; Yu et al. 2016).

As these studies were based on repeated cross-sectional designs, they were unable to determine to what extent the observed cohort trends result from differences in levels of disability  present as people reach old age (e.g., accumulated earlier in life) and/or changing disability trajectories during old age itself (e.g., accelerated decline). To answer these questions, we need longitudinal analyses following cohorts over time.

Dynamic cohort studies are used to assess whether the disability trajectory of any given cohort is shaped by social and demographic forces. As the nature of these forces changes, cohort-specific health trajectories are also likely to change. Hence, comparative analyses of the health trajectories of consecutive cohorts provide novel insights into the dynamics behind aggregate level health trends. Few studies of this type have been conducted to explore sex and cohort-specific trajectories of functional decline among older adults. One exception is a study based on community dwelling older adults in the UK, in which declines in the prevalence of functional impairments were observed among men but not women (Morciano et al. 2015). A few studies have explored cohort-specific trajectories of frailty in old age. One revealed a pessimistic pattern, where later born British cohorts had higher prevalence of frailty than earlier cohorts upon entering old age, while the rate of decline during old age was similar for all (Marshall et al. 2015). American data also showed that later born cohorts of older adults have greater prevalence of frailty and self-reported illness than preceding cohorts (Mirowsky and Ross 2008; Yang and Lee 2009, 2010).

In sum, the evidence on cohort differences in old-age disabilities is scarce and the findings are ambiguous. With this study, we will extend current understanding of cohort-specific disability trajectories for women and men in different European regions. Our aim is to provide evidence of how disability trajectories in old age differ depending on birth cohort, sex and geopolitical context.

## Data and methods

The Survey of Health, Ageing and Retirement in Europe (SHARE) is a cross-national and longitudinal survey that collects data on health, social and economic factors among Europeans aged 50 and older (Börsch-Supan et al. 2013). Our sample includes data collected on seven different occasions (survey waves) between 2004 and 2017 by computer-assisted face-to-face interviews. The sampling procedures differ between the countries, and several different methods have been used to calculate and compare response rates. Across different methods, household response rates range between 32 and 96% for the first survey (Wave 1) (Bergmann et al. 2019).

In order to analyze the trajectories of subsequent birth cohorts, we have construed an analytic sample consisting of five five-year cohorts born between 1920 and 1944 from all European countries that participated in the first or second wave of the SHARE data collection. Due to limited national sample sizes and, consequently, lack of statistical power in the older age groups, we have aggregated the thirteen included countries into the following four region-based groups:

- *Northern Europe* Sweden and Denmark.
- *Western Europe* Austria, Germany, the Netherlands, France, Switzerland, and Belgium.
- *Southern Europe* Spain, Italy, and Greece.
- *Eastern Europe* Czech Republic and Poland.

These groupings are based on the welfare state typology developed by Ferrera (1996), albeit with two modifications. We were not able to include the Anglo-Saxon group due to lack of data. On the other hand, we expanded the typology by adding a group for Eastern Europe (as has previously been done by Eikemo et al. (2008)). This typology is initially based on differences in social policies, as indicated by coverage, replacement rates and poverty rates (Bambra 2007; Ferrera 1996). Besides similarities in terms of social policies, the groups are also characterized by regional proximity. These four groups (albeit with some differences in the included countries, depending on the waves included) have been used in previous studies that have examined physical functioning based on SHARE data (Ahrenfeldt et al. 2018, 2019; Scheel-Hincke et al. 2020). These studies have revealed different patterns and trends across the regional groups.

## Variables

To assess disabilities, we created indices of self-reported limitations in activities of daily living (ADL) and instrumental activities of daily living (IADL) (Ahrenfeldt et al. 2018). The ADL index consisted of six tasks that assess whether the respondent had difficulties with: dressing; bathing or showering; eating or cutting up food; walking across a room; and using the toilet including getting up or down. The IADL index included seven items that assessed whether respondents had difficulties with: using a map in a unknown place; preparing a hot meal; shopping for groceries; making a telephone call; taking medications; doing work around the house or garden; and managing money. From these indices, two binary variables that indicated whether the respondents reported ADL or IADL limitations were created. For each measure of disability, the respective binary variable was coded 0 if respondents had no limitations, and 1 if the respondent had at least one limitation. The rationale for dichotomizing the variables is that *any* ADL or IADL limitation is an indication of need for help and support. That is, the dichotomized variables are meant to be indicators of autonomy or dependence rather than of severity of disabilities.

## Analytic strategy

To analyze trajectories of physical functioning within cohorts, we structured the data as repeated observations for individuals that initially participated in SHARE wave 1 (2004) or wave 2 (2007) and at least one subsequent panel wave. We fit mixed effects logistic regression models that estimated the level of the binary indicator for ADL and IADL limitations at baseline (wave 1 or wave 2) and then the change in ADL and IADL limitations through subsequent waves up to wave 7 (2017) for five-year cohorts from each region. Mixed effect regression methods are suitable for handling repeated measures of the same individuals as well as unbalanced panel data. A similar analytical strategy has previously been used in studies that examined cohort trajectories of frailty in the UK (Marshall et al. 2015; Rogers et al. 2017).

The mixed effects logistic regression model included effects for survey wave (time), cohort, and sex (Model 1). The model allowed for random intercepts at baseline and random slopes. Interaction terms were included between sex and cohort, and between wave and cohort, these interaction terms allowed us to model different slopes across waves and between sexes. We tested a quadratic wave term to allow the individual slopes to take nonlinear shapes; however, the quadratic term was not statistically significant and did not alter the model substantially and consequently was not included in the final model. The model was estimated separately for the two outcomes ADL and IADL limitations in subsamples of the four European regions specified above. In a second step, the models were additionally adjusted for education (classified according to ISCED 1997) and household income, to assess to what extent the observed patterns could be attributed to socioeconomic conditions (Model 2). Household income was adjusted for the number of household members by dividing the total household income by the square root of the number of household members (Martin 2017).

**Fig. 1:**
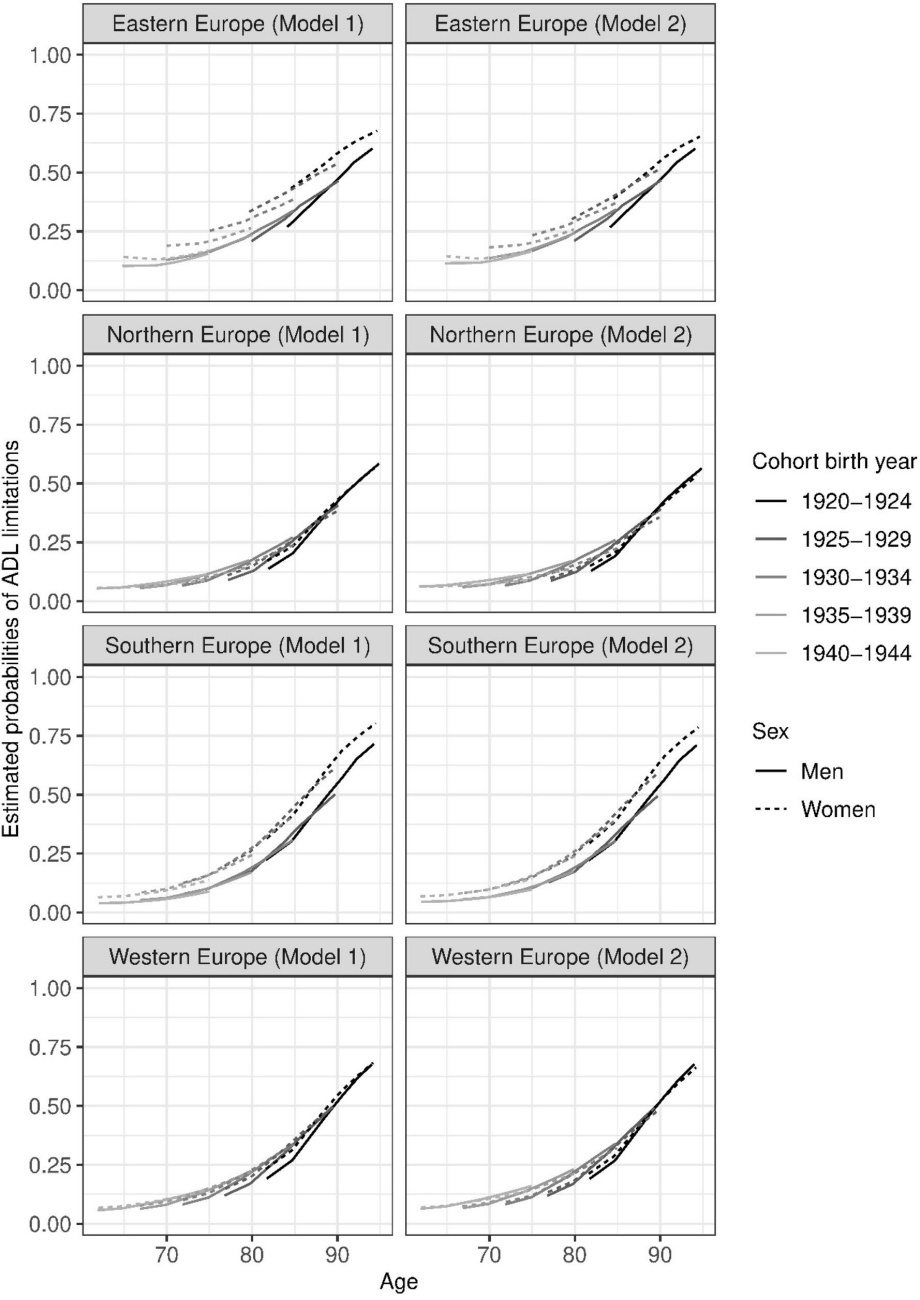
Estimated Probabilities of ADL limitations in European regions, 2004–2017 without (Model 1) and with adjustments for education and income (Model 2). Estimated from mixed effects logistic regression models, see Supplementary Tables 1–10

**Fig. 2:**
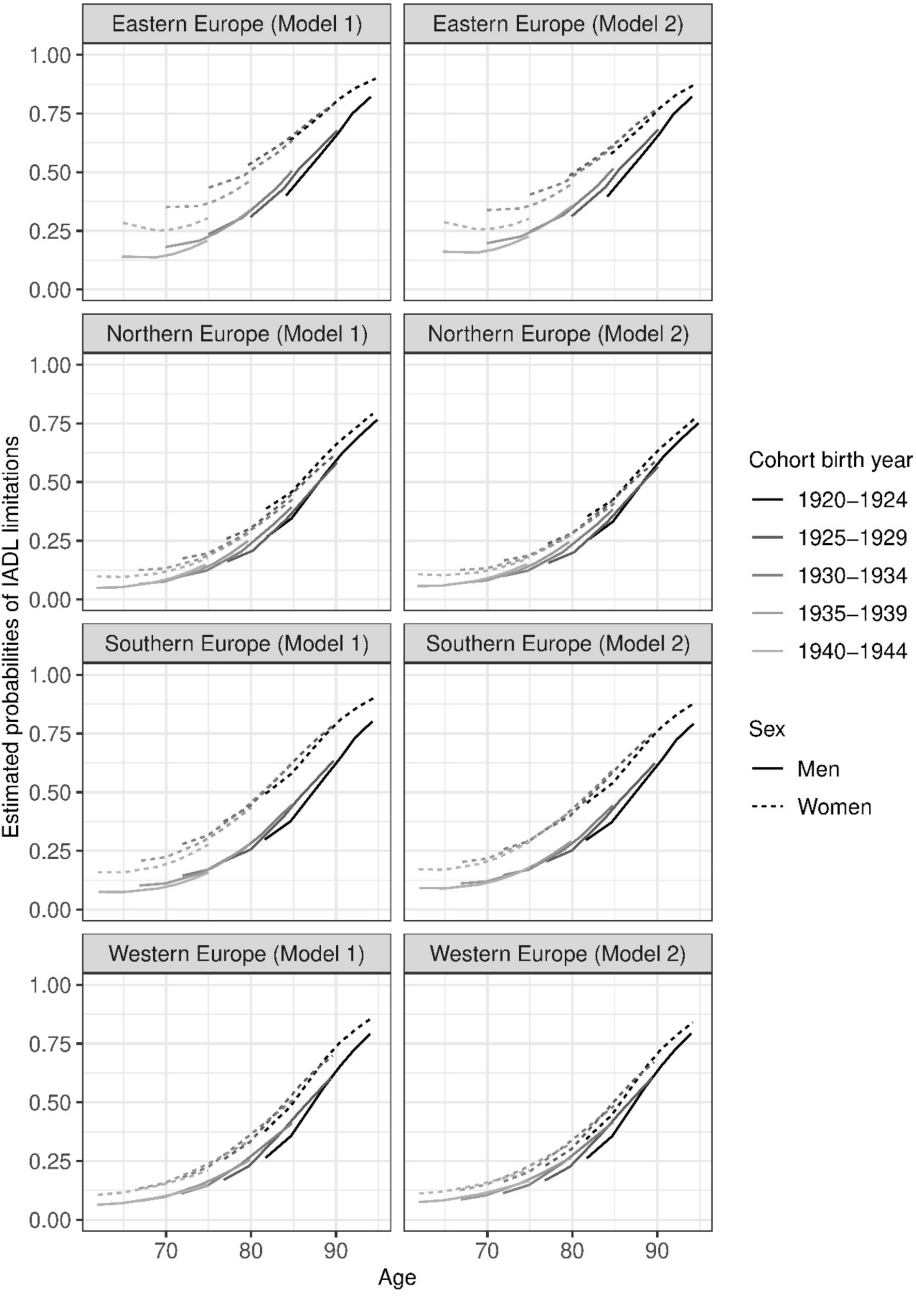
Estimated Probabilities of ADL limitations in European regions, 2004–2017 without (Model 1) and with adjustments for education and income (Model 2). Estimated from mixed effects logistic regression models, see Supplementary Tables 1, 2 and 11–18

From these models, we estimated the probability of the outcome occurring for each cohort across waves and by sex, using the *margins* command in Stata version 14.2. In Figs. 1–2, wave (time) was replaced by the average age of each cohort at each survey wave to facilitate a more intuitive interpretation of the growth in the outcome measure over time.

In analyses, we used calibrated cross-sectional weights from the first wave in which a participant was included (Malter and Börsch-Supan 2013).

## Results

Table 1 shows descriptive statistics for the complete pooled data from all regions, by cohort and wave. The number of observations in each cohort at baseline ranged from 1757 men and 2031 women in the youngest cohort (born between 1940 and 1944), to 464 men and 682 women in the oldest cohort (born between 1920 and 1924). For each cohort, the highest number of observations was seen in wave two. This occurred because we included respondents that entered the survey at either wave one or wave two.

**Table 1:** Descriptive statistics of the analytical sample, all 13 included countries pooled

Cohort		Survey wave, year					
		Wave 1	Wave 2	Wave 4	Wave 5	Wave 6	Wave 7
		2004	2007	2011	2013	2015	2017
1940–1944	Men (*n*)	1757	2263	1588	1494	1463	1319
	Women (*n*)	2031	2662	1846	1774	1769	1633
	Mean age (years)	61.9	64.9	68.9	70.9	72.9	74.9
	ADL limitations (%)	7.1	7.7	8.6	10.0	10.8	11.9
	IADL limitations (%)	13.2	15.3	15.8	18.4	21.6	22.6
1935–1939	Men (*n*)	1634	2076	1383	1282	1267	1069
	Women (*n*)	1788	2231	1520	1455	1451	1261
	Mean age (years)	66.9	69.9	73.9	75.9	77.9	79.9
	ADL limitations (%)	6.9	9.6	14.0	15.9	17.6	19.5
	IADL limitations (%)	17.6	21.0	25.3	28.5	34.1	40.7
1930–1934	Men (*n*)	1269	1585	1038	953	859	693
	Women (*n*)	1442	1759	1169	1117	1087	865
	Mean age (years)	71.9	74.9	78.9	80.9	82.8	84.8
	ADL limitations (%)	11.6	15.3	22.0	23.5	27.0	30.8
	IADL limitations (%)	24.7	31.8	35.8	43.2	48.9	56.0
1925–1929	Men (*n*)	870	1013	591	475	390	265
	Women (*n*)	1127	1331	863	746	666	464
	Mean age (years)	76.9	79.9	83.8	85.7	87.7	89.7
	ADL limitations (%)	16.7	21.8	30.1	36.1	43.8	46.1
	IADL limitations (%)	31.5	38.6	48.0	56.8	66.7	71.8
1920–1924	Men (*n*)	464	520	256	189	133	69
	Women (*n*)	682	760	383	302	218	136
	Mean age (years)	81.7	84.7	88.6	90.5	92.4	94.3
	ADL limitations (%)	27.1	33.8	48.6	54.0	56.0	61.8
	IADL limitations (%)	43.8	53.0	70.5	70.5	77.5	80.1

ADL and IADL limitations increased with age within each cohort in our sample. In the first wave (2004) of the youngest cohort (aged between 60 and 64) 7.1 percent had ADL limitations, while at the last observation (wave 7) when the cohort was aged between 73 and 77, this proportion had increased to 11.9 percent. The corresponding increase in IADL limitations ranged from 13.2 percent in wave one to 22.6 in wave seven. In the oldest cohort, aged 80 to 84 (born between 1920 and 1924) at wave one, 27.1 percent had ADL limitations, whereas by the end of the follow-up period this proportion had reached 61.8 percent of those then aged 93 to 97. The corresponding increase in IADL limitations in this cohort ranged from 43.8 percent in wave 1 to 80.1 in wave 7.

Figures 1 and 2 show the estimated probabilities of ADL and IADL for each of the four European regions, without and with adjustments for education and income. The underlying mixed effects logistic regression models that generated the estimated probabilities are presented in Supplementary Tables 1, 2 and 3. Estimations per cohort, age and sex are available in Supplementary Tables 4–10 (Fig. 1), and 11–18 (Fig. 2) and 10–13.

Overall trends in ADL limitations were similar in all four regions, albeit with some differences in prevalence levels. In Eastern Europe, initial levels of ADL limitations in the latest born cohorts were higher than elsewhere. In the earliest cohorts, the highest prevalence of ADL limitations was observed in Southern and Western Europe.

The prevalence of ADL limitations increased with age in each cohort (Fig. 1). However, the age trends across cohorts differed depending on sex and region. Among men in Eastern, Northern and Western Europe, and for women in Northern and Western Europe, earlier cohorts tended to have a lower prevalence of ADL limitations than did later cohorts at the same ages. The reversed pattern was observed for women in Eastern Europe, where earlier cohorts showed a higher prevalence of ADL limitations than later cohorts. In Southern Europe, the age pattern of ADL limitations overlapped almost completely across cohorts.

We also observed variations in sex differences in the patterning of ADL limitations across regions. In Northern and Western Europe, sex differences in ADL limitations were small or non-existent in all age groups. In Eastern and Southern Europe, there were marked sex differences in ADL limitations with women reporting more limitations than men. In Eastern Europe, sex differences were greater in earlier cohorts. In Southern Europe, there were no discernible cohort effects in the sex differences across cohorts. In Eastern, Northern and Western Europe the sex differences tended to decrease with age within each cohort, whereas the opposite pattern was observed in Southern Europe (Fig. 1).

Overall, trends in IADL limitations were similar to those for ADL limitations (Fig. 2). Here too we observed a higher prevalence of limitations among more recent cohorts in Eastern Europe compared to other regions. For earlier cohorts (born 1920–1924 and 1925–1929), regional differences were small, and only Northern Europe showed a somewhat lower prevalence of IADL limitations compared to other regions.

Only men in Eastern and Western Europe showed trends toward higher rates of limitations in more recent cohorts. Among all women in Eastern Europe and the two most recent cohorts in Southern Europe, the reverse pattern emerged. Here, each subsequent more recent birth cohort had a substantially lower prevalence of limitations compared with earlier cohorts.

In all age groups and in all regions, women had higher prevalence of IADL limitations than men. The sex differences in IADL limitations (Fig. 2) were more marked than in ADL limitations (Fig. 1). Again, the greatest differences between men and women were seen in Eastern and Southern Europe although these were also present in Northern and Western Europe, but at lower levels (Fig. 2).

In general, adjusting for education and income (Model 2) did not change the overall patterns but did attenuated somewhat the sex and cohort differences.

## Discussion

In this study, we followed the disability trajectories of women and men from five subsequent birth cohorts and four European regions, over a period of 13 years. Perhaps most disconcerting were the increasing probabilities of disabilities observed across subsequent birth cohorts, especially among men in Eastern, Northern and Western Europe, but also among women in Northern and Western Europe.

Overall, findings varied by cohort, region and sex. There were sex differences in ADL and, particularly in IADL limitations in all regions for most cohorts. Women reported more limitations than men. However, these sex differences were more marked in Eastern and Southern than Northern and Western Europe. Overall, sex differences in prevalence of functional limitations tended to decrease with age within birth cohorts. Among men in Eastern, Northern and Western Europe later born cohorts tended to report disabilities more than did earlier birth cohorts at the same ages. A similar pattern was observed for women in Northern and Western Europe. In contrast, the risk of disabilities was lower in later born cohorts than in previous birth cohorts among women in Eastern Europe.

Adjusting for education and income attenuated the observed sex and cohort difference somewhat, but not entirely.

These results should be interpreted with caution. This is a descriptive study, and as such interpretation of the results depends on whether samples are representative. In our study design, there are two major threats to this assumption. First, not all countries in Europe participated in earlier waves of SHARE, which means that we rely on data only from a non-random subset of countries. This poses limitations on inferences drawn from results. For example, in this study Eastern Europe is represented by only two countries: Czech Republic and Poland. To the extent the patterns differ for other countries in the region, our results cannot be generalized to the whole of Eastern Europe. Second, non-response and attrition rates of the SHARE survey threaten representativeness. The response rate for our baseline, Wave 1 of SHARE, ranged between 51 and 67 percent for the countries included in analyses. As non-respondents are likely to be a selected group in terms of health and health-related characteristics, this may bias results. Moreover, due to the longitudinal design in this study we included only respondents that had participated in two or more survey waves, further exacerbating the non-response bias. To minimize the impact of non-response on the estimates, we used calibrated cross-sectional weights provided by SHARE to compensate for selective non-response.

On the other hand, SHARE data offer a rich source of longitudinal information that allowed disaggregation of observed cohort patterns by sex and region. Taken together with the novel application of linear mixed methods for tracking cohort-specific health trajectories, pioneered by Marshall et al. (2015), we were able to distinguish broad, dynamic, patterns of old-age disability trajectories across subsequent cohorts of older Europeans.

The observed increase in disabilities in later born cohorts in the Nordic countries is seemingly at odds with previous repeated cross-section studies, that have documented decreasing prevalence of disabilities (Ahrenfeldt et al. 2018; Christensen et al. 2013; Hossin et al. 2019). Yet, the findings are in line with a cohort study which found increasing rates of disabilities in later born cohorts in most European countries, including the Nordic (Beller and Epping 2021). This discrepancy underscores the difference between studying health trends through repeated cross-sections and through cohort succession. Results suggest that the positive development observed in previous studies is unlikely to be explained by younger cohorts entering old age with better function. Rather the explanation more likely depends on development of disabilities within cohorts (where we see a steeper increase in the prevalence of disabilities with age among earlier cohorts than later) or on the age structure of the entire older population.

The greatest sex differences in disabilities were observed in Eastern and Southern Europe. This is largely in line with the findings for the age groups 65 + from an earlier study of sex differences in old-age ADL and IADL limitations in Europe (Scheel-Hincke et al. 2020). Van Oyen et al. (2010) found that, at a societal level, the sex-gap in disability-free life expectancy was positively associated with gross domestic product, expenditure on elder care and income differences between women and men, as these factors are more favourable in Northern and Western Europe than in Eastern and Southern Europe. These factors may also contribute to the patterns observed in this study. In line with this hypothesis, the sex differences in disabilities decreased in our models once we adjusted for education and income. It is also quite possible that these macro-level factors played a role in shaping the observed cohort differences.

The strongest cohort differences were observed among more recent cohorts of women in Eastern Europe, with each subsequent cohort reporting less disability than the previous. These differences could be linked with rapid and sizeable changes in the living conditions for older people that countries in this region experienced after the fall of communism. This is a positive development both because it foreshadows a decreasing future burden of later-life disabilities in Eastern Europe and a diminishing sex gap in the likelihood of disabilities in older adults.

Overall, the results from this study suggest that disability trajectories in different cohorts of men and women were by and large similar across Europe. The trajectories varied more depending on sex, age, and region than depending on cohort. Future studies on trends in old-age disabilities might focus on mapping out and explaining sex and geographical inequalities in health.

In addition, the increasing prevalence of disabilities among later born cohorts observed in Eastern, Northern and Western Europe warrants attention. Beller and Epping (2021) suggested three different hypotheses that could contribute to this pattern. It might be a consequence of increasing survival among individuals with health problems (‘sick survivors’) as suggested by the ‘failures of success’ hypothesis (Gruenberg 1977; Kingston et al. 2017), or of changing behavioral patterns and living conditions in later born cohorts that make them more susceptible to disabilities (e.g., obesity, sedentary lifestyles, labour market conditions). Finally, there may be cohort differences in the perception and likelihood of reporting disabilities due to different cognitive ‘benchmarks’ established throughout the life course of what constitutes a disability. Future studies should test validity of these hypotheses empirically, as they imply different policy responses.

## Supplementary Information

Below is the link to the electronic supplementary material.

Supplementary file1 (DOCX 338 KB)

## Data Availability

SHARE data are readily available throught the SHARE homepage (http://www.share-project.org/home0.html).

## Code availability

The code used for the analyses is available at request.
