# Supplementary material for: Cohort-specific disability trajectories among older women and men in Europe 2004–2017
Source: Eur J Ageing. 2022 Feb 19;19(4):1111–9. doi: 10.1007/s10433-022-00684-4 (PMC9729672; doi:10.1007/s10433-022-00684-4)
Supplement: Supplementary file 1 — Supplementary file1 (DOCX 338 KB) [file 10433_2022_684_MOESM1_ESM.docx]

**Supplementary data**

**Study**Cohort trajectories of functional decline among older women and men in Europe 2004-2017

**Content**

Supplementary table 1–18

**Supplementary table 1.** Mixed effects logistic regression. Outcome: ADL and IADL. Stratified by region, **model 1** (Figure 1 and Figure 2).

|  | ADL |  |  |  |  | IADL |  |  |  | |
| --- | --- | --- | --- | --- | --- | --- | --- | --- | --- | --- |
|  | Odds ratio | P-value | LCI | UCI |  | Odds ratio | P-value | LCI | UCI | |
| **Northern Europe**  Observations: 11429  Individuals: 2829 |  |  |  |  |  |  |  |  |  | |
| **Fixed effects** |  |  |  |  |  |  |  |  |  | |
| Sex (1=women) | 1.394 | 0.176 | 0.862 | 2.255 |  | 3.022 | 0.000 | 1.992 | 4.586 | |
| Cohort | 1.224 | 0.070 | 0.983 | 1.522 |  | 1.793 | 0.000 | 1.464 | 2.197 | |
| Wave | 1.430 | 0.003 | 1.130 | 1.813 |  | 1.200 | 0.085 | 0.975 | 1.476 | |
| Wave*cohort | 1.184 | 0.000 | 1.129 | 1.242 |  | 1.174 | 0.000 | 1.123 | 1.226 | |
| Sex*wave | 0.907 | 0.074 | 0.815 | 1.010 |  | 0.900 | 0.032 | 0.819 | 0.991 | |
| Sex*cohort | 1.088 | 0.448 | 0.875 | 1.354 |  | 0.975 | 0.805 | 0.798 | 1.191 | |
| Wave*wave | 0.994 | 0.677 | 0.968 | 1.022 |  | 1.033 | 0.008 | 1.008 | 1.058 | |
| Cohort*cohort | 1.117 | 0.022 | 1.016 | 1.229 |  | 1.112 | 0.015 | 1.021 | 1.210 | |
| Intercept | 0.010 | 0.000 | 0.005 | 0.018 |  | 0.021 | 0.000 | 0.012 | 0.036 | |
| **Random effects** |  |  |  |  |  |  |  |  |  | |
| Wave | 1.164 |  | 1.105 | 1.261 |  | 1.151 |  | 1.100 | 1.231 | |
| Intercept | 113.863 |  | 37.077 | 495.219 |  | 124.711 |  | 45.331 | 448.990 | |
|  |  |  |  |  |  |  |  |  |  | |
| **Western Europe**  Observations: 27732  Individuals: 7347 |  |  |  |  |  |  |  |  |  | |
| **Fixed effects** |  |  |  |  |  |  |  |  |  | |
| Sex (1=women) | 1.489 | 0.036 | 1.027 | 2.158 |  | 2.286 | 0.000 | 1.684 | 3.108 | |
| Cohort | 1.443 | 0.000 | 1.204 | 1.732 |  | 1.578 | 0.000 | 1.351 | 1.842 | |
| Wave | 1.545 | 0.000 | 1.293 | 1.844 |  | 1.454 | 0.000 | 1.239 | 1.706 | |
| Wave*cohort | 1.163 | 0.000 | 1.116 | 1.210 |  | 1.174 | 0.000 | 1.134 | 1.214 | |
| Sex*wave | 0.944 | 0.212 | 0.862 | 1.034 |  | 1.003 | 0.945 | 0.931 | 1.080 | |
| Sex*cohort | 1.043 | 0.657 | 0.865 | 1.259 |  | 1.029 | 0.724 | 0.878 | 1.206 | |
| Wave*wave | 0.997 | 0.801 | 0.976 | 1.018 |  | 1.007 | 0.491 | 0.988 | 1.026 | |
| Cohort*cohort | 1.126 | 0.002 | 1.044 | 1.215 |  | 1.099 | 0.004 | 1.030 | 1.171 | |
| Intercept | 0.011 | 0.000 | 0.007 | 0.017 |  | 0.024 | 0.000 | 0.017 | 0.035 | |
| **Random effects** |  |  |  |  |  |  |  |  |  | |
| Wave | 1.130 |  | 1.085 | 1.200 |  | 1.089 |  | 1.055 | 1.142 | |
| Intercept | 194.028 |  | 77.478 | 589.928 |  | 77.401 |  | 40.085 | 168.174 | |
|  |  |  |  |  |  |  |  |  |  |  |
| **Southern Europe**  Observations: 17785  Individuals: 4622 |  |  |  |  |  |  |  |  |  |  |
| **Fixed effects** |  |  |  |  |  |  |  |  |  |  |
| Sex (1=women) | 1.978 | 0.001 | 1.338 | 2.921 |  | 3.040 | 0.000 | 2.273 | 4.063 |  |
| Cohort | 1.679 | 0.000 | 1.380 | 2.040 |  | 1.510 | 0.000 | 1.300 | 1.754 |  |
| Wave | 1.328 | 0.005 | 1.091 | 1.616 |  | 1.127 | 0.123 | 0.969 | 1.314 |  |
| Wave*cohort | 1.181 | 0.000 | 1.132 | 1.231 |  | 1.149 | 0.000 | 1.112 | 1.188 |  |
| Sex*wave | 1.039 | 0.390 | 0.952 | 1.132 |  | 1.027 | 0.430 | 0.961 | 1.097 |  |
| Sex*cohort | 1.022 | 0.822 | 0.841 | 1.244 |  | 1.035 | 0.649 | 0.892 | 1.201 |  |
| Wave*wave | 1.007 | 0.544 | 0.984 | 1.031 |  | 1.029 | 0.002 | 1.010 | 1.049 |  |
| Cohort*cohort | 1.104 | 0.014 | 1.020 | 1.196 |  | 1.044 | 0.156 | 0.984 | 1.107 |  |
| Intercept | 0.017 | 0.000 | 0.010 | 0.026 |  | 0.070 | 0.000 | 0.050 | 0.096 |  |
| **Random effects** |  |  |  |  |  |  |  |  |  |  |
| Wave | 1.108 |  | 1.071 | 1.168 |  | 1.070 |  | 1.048 | 1.103 |  |
| Intercept | 57.743 |  | 27.827 | 140.611 |  | 12.936 |  | 8.432 | 21.628 |  |
|  |  |  |  |  |  |  |  |  |  |  |
| **Eastern Europe**  Observations: 5047  Individuals: 1373 |  |  |  |  |  |  |  |  |  |  |
| **Fixed effects** |  |  |  |  |  |  |  |  |  |  |
| Sex (1=women) | 2.171 | 0.019 | 1.134 | 4.154 |  | 3.770 | 0.000 | 2.016 | 7.057 |  |
| Cohort | 1.044 | 0.808 | 0.739 | 1.474 |  | 1.164 | 0.327 | 0.860 | 1.576 |  |
| Wave | 0.880 | 0.544 | 0.581 | 1.332 |  | 0.922 | 0.647 | 0.652 | 1.303 |  |
| Wave*cohort | 1.168 | 0.000 | 1.088 | 1.254 |  | 1.165 | 0.000 | 1.095 | 1.240 |  |
| Sex*wave | 0.956 | 0.559 | 0.820 | 1.113 |  | 0.945 | 0.393 | 0.829 | 1.077 |  |
| Sex*cohort | 1.138 | 0.414 | 0.834 | 1.553 |  | 1.041 | 0.770 | 0.795 | 1.363 |  |
| Wave*wave | 1.028 | 0.223 | 0.983 | 1.077 |  | 1.046 | 0.019 | 1.007 | 1.087 |  |
| Cohort*cohort | 1.021 | 0.711 | 0.913 | 1.142 |  | 1.027 | 0.618 | 0.923 | 1.145 |  |
| Intercept | 0.143 | 0.000 | 0.055 | 0.368 |  | 0.204 | 0.000 | 0.091 | 0.457 |  |
| **Random effects** |  |  |  |  |  |  |  |  |  |  |
| Wave | 1.191 |  | 1.126 | 1.294 |  | 1.069 |  | 1.033 | 1.150 |  |
| Intercept | 2.404 |  | 1.318 | 16.184 |  | 5.058 |  | 2.509 | 17.392 |  |

*Note.* LCI, lower confidence interval. UCI, upper confidence interval.

**Supplementary table 2.** Mixed effects logistic regression. Outcome: ADL and IADL. Stratified by region, **model 2**, adjusted for education and income (Figure 1 and Figure 2).

|  | ADL |  |  |  |  | IADL |  |  |  | |
| --- | --- | --- | --- | --- | --- | --- | --- | --- | --- | --- |
|  | Odds ratio | P-value | LCI | UCI |  | Odds ratio | P-value | LCI | UCI | |
| **Northern Europe**  Observations: 11429  Individuals: 2829 |  |  |  |  |  |  |  |  |  | |
| **Fixed effects** |  |  |  |  |  |  |  |  |  | |
| Sex (1=women) | 1.251 | 0.357 | 0.776 | 2.016 |  | 2.707 | 0.000 | 1.789 | 4.097 | |
| Cohort | 1.126 | 0.287 | 0.905 | 1.401 |  | 1.640 | 0.000 | 1.341 | 2.006 | |
| Wave | 1.439 | 0.003 | 1.131 | 1.830 |  | 1.191 | 0.102 | 0.966 | 1.469 | |
| Wave*cohort | 1.186 | 0.000 | 1.131 | 1.245 |  | 1.178 | 0.000 | 1.127 | 1.231 | |
| Sex*wave | 0.911 | 0.088 | 0.819 | 1.014 |  | 0.904 | 0.039 | 0.822 | 0.995 | |
| Sex*cohort | 1.071 | 0.537 | 0.863 | 1.329 |  | 0.957 | 0.666 | 0.785 | 1.167 | |
| Wave*wave | 0.991 | 0.531 | 0.964 | 1.019 |  | 1.030 | 0.015 | 1.006 | 1.056 | |
| Cohort*cohort | 1.129 | 0.012 | 1.027 | 1.240 |  | 1.122 | 0.007 | 1.032 | 1.220 | |
| Household income | 1.000 | 0.000 | 1.000 | 1.000 |  | 1.000 | 0.000 | 1.000 | 1.000 | |
| Education | 0.855 | 0.002 | 0.774 | 0.945 |  | 0.864 | 0.002 | 0.789 | 0.946 | |
| Intercept | 0.024 | 0.000 | 0.012 | 0.045 |  | 0.050 | 0.000 | 0.027 | 0.090 | |
| **Random effects** |  |  |  |  |  |  |  |  |  | |
| Wave | 1.158 |  | 1.100 | 1.254 |  | 1.150 |  | 1.100 | 1.229 | |
| Intercept | 93.137 |  | 31.886 | 379.122 |  | 93.558 |  | 36.127 | 311.855 | |
|  |  |  |  |  |  |  |  |  |  | |
| **Western Europe**  Observations: 27732  Individuals: 7347 |  |  |  |  |  |  |  |  |  | |
| **Fixed effects** |  |  |  |  |  |  |  |  |  | |
| Sex (1=women) | 1.284 | 0.187 | 0.886 | 1.862 |  | 1.885 | 0.000 | 1.388 | 2.559 | |
| Cohort | 1.384 | 0.000 | 1.155 | 1.658 |  | 1.485 | 0.000 | 1.275 | 1.731 | |
| Wave | 1.555 | 0.000 | 1.303 | 1.856 |  | 1.465 | 0.000 | 1.249 | 1.719 | |
| Wave*cohort | 1.160 | 0.000 | 1.115 | 1.208 |  | 1.173 | 0.000 | 1.134 | 1.213 | |
| Sex*wave | 0.938 | 0.163 | 0.857 | 1.026 |  | 0.995 | 0.891 | 0.924 | 1.072 | |
| Sex*cohort | 1.025 | 0.793 | 0.852 | 1.233 |  | 1.007 | 0.930 | 0.863 | 1.175 | |
| Wave*wave | 0.997 | 0.773 | 0.976 | 1.018 |  | 1.006 | 0.525 | 0.987 | 1.026 | |
| Cohort*cohort | 1.146 | 0.000 | 1.062 | 1.236 |  | 1.125 | 0.000 | 1.056 | 1.198 | |
| Household income | 1.000 | 0.001 | 1.000 | 1.000 |  | 1.000 | 0.001 | 1.000 | 1.000 | |
| Education | 0.764 | 0.000 | 0.704 | 0.829 |  | 0.701 | 0.000 | 0.654 | 0.750 | |
| Intercept | 0.027 | 0.000 | 0.017 | 0.043 |  | 0.072 | 0.000 | 0.049 | 0.107 | |
| **Random effects** |  |  |  |  |  |  |  |  |  | |
| Wave | 1.125 |  | 1.081 | 1.193 |  | 1.085 |  | 1.054 | 1.136 | |
| Intercept | 153.953 |  | 64.473 | 440.929 |  | 56.656 |  | 30.591 | 117.249 | |
|  |  |  |  |  |  |  |  |  |  |  |
| **Southern Europe**  Observations: 17785  Individuals: 4622 |  |  |  |  |  |  |  |  |  |  |
| **Fixed effects** |  |  |  |  |  |  |  |  |  |  |
| Sex (1=women) | 1.738 | 0.005 | 1.180 | 2.559 |  | 2.526 | 0.000 | 1.904 | 3.350 |  |
| Cohort | 1.600 | 0.000 | 1.311 | 1.954 |  | 1.403 | 0.000 | 1.206 | 1.632 |  |
| Wave | 1.332 | 0.004 | 1.095 | 1.620 |  | 1.115 | 0.164 | 0.957 | 1.300 |  |
| Wave*cohort | 1.177 | 0.000 | 1.129 | 1.226 |  | 1.148 | 0.000 | 1.111 | 1.187 |  |
| Sex*wave | 1.038 | 0.393 | 0.953 | 1.131 |  | 1.029 | 0.399 | 0.963 | 1.099 |  |
| Sex*cohort | 1.012 | 0.904 | 0.832 | 1.231 |  | 1.018 | 0.816 | 0.879 | 1.178 |  |
| Wave*wave | 1.007 | 0.575 | 0.983 | 1.030 |  | 1.030 | 0.002 | 1.011 | 1.049 |  |
| Cohort*cohort | 1.128 | 0.003 | 1.043 | 1.221 |  | 1.072 | 0.018 | 1.012 | 1.136 |  |
| Household income | 1.000 | 0.220 | 1.000 | 1.000 |  | 1.000 | 0.016 | 1.000 | 1.000 |  |
| Education | 0.670 | 0.000 | 0.589 | 0.761 |  | 0.583 | 0.000 | 0.527 | 0.645 |  |
| Intercept | 0.031 | 0.000 | 0.019 | 0.050 |  | 0.171 | 0.000 | 0.123 | 0.240 |  |
| **Random effects** |  |  |  |  |  |  |  |  |  |  |
| Wave | 1.105 |  | 1.069 | 1.160 |  | 1.073 |  | 1.050 | 1.105 |  |
| Intercept | 48.005 |  | 23.824 | 112.922 |  | 8.712 |  | 5.912 | 13.974 |  |
|  |  |  |  |  |  |  |  |  |  |  |
| **Eastern Europe**  Observations: 5047  Individuals: 1373 |  |  |  |  |  |  |  |  |  |  |
| **Fixed effects** |  |  |  |  |  |  |  |  |  |  |
| Sex (1=women) | 1.868 | 0.059 | 0.977 | 3.571 |  | 3.072 | 0.000 | 1.657 | 5.695 |  |
| Cohort | 1.018 | 0.917 | 0.725 | 1.430 |  | 1.119 | 0.455 | 0.834 | 1.501 |  |
| Wave | 0.910 | 0.659 | 0.600 | 1.381 |  | 0.960 | 0.819 | 0.680 | 1.356 |  |
| Wave*cohort | 1.163 | 0.000 | 1.084 | 1.249 |  | 1.159 | 0.000 | 1.091 | 1.231 |  |
| Sex*wave | 0.950 | 0.513 | 0.816 | 1.107 |  | 0.936 | 0.317 | 0.823 | 1.065 |  |
| Sex*cohort | 1.108 | 0.515 | 0.814 | 1.506 |  | 1.012 | 0.929 | 0.781 | 1.310 |  |
| Wave*wave | 1.026 | 0.266 | 0.981 | 1.074 |  | 1.042 | 0.032 | 1.004 | 1.081 |  |
| Cohort*cohort | 1.028 | 0.623 | 0.921 | 1.148 |  | 1.032 | 0.544 | 0.931 | 1.145 |  |
| Household income | 1.000 | 0.967 | 1.000 | 1.000 |  | 1.000 | 0.605 | 1.000 | 1.000 |  |
| Education | 0.746 | 0.000 | 0.636 | 0.875 |  | 0.672 | 0.000 | 0.587 | 0.770 |  |
| Intercept | 0.250 | 0.007 | 0.091 | 0.686 |  | 0.435 | 0.045 | 0.193 | 0.982 |  |
| **Random effects** |  |  |  |  |  |  |  |  |  |  |
| Wave | 1.179 |  | 1.115 | 1.283 |  | 1.055 |  | 1.024 | 1.127 |  |
| Intercept | 2.461 |  | 1.311 | 19.899 |  | 4.850 |  | 2.494 | 15.312 |  |

*Note.* LCI, lower confidence interval. UCI, upper confidence interval.

**Supplementary table 3.** Predicted probabilities of **ADL** limitations by age and sex (**model 1**). **Eastern Europe**, data for figure 1. (PP, predicted probability. LCI, lower confidence interval. UCI, upper confidence interval).

|  |  | Cohort 1940-1944 | | | Cohort 1935-1939 | | | Cohort 1930-1934 | | | Cohort 1925-1929 | | | Cohort 1920-1924 | | |
| --- | --- | --- | --- | --- | --- | --- | --- | --- | --- | --- | --- | --- | --- | --- | --- | --- |
| Age | Sex | PP | LCI | UCI | PP | LCI | UCI | PP | LCI | UCI | PP | LCI | UCI | PP | LCI | UCI |
| … |  |  |  |  |  |  |  |  |  |  |  |  |  |  |  |  |
| 65 | M | 0.103 | 0.063 | 0.143 |  |  |  |  |  |  |  |  |  |  |  |  |
| 65 | W | 0.142 | 0.096 | 0.187 |  |  |  |  |  |  |  |  |  |  |  |  |
| 66 | M |  |  |  |  |  |  |  |  |  |  |  |  |  |  |  |
| 66 | W |  |  |  |  |  |  |  |  |  |  |  |  |  |  |  |
| 67 | M |  |  |  |  |  |  |  |  |  |  |  |  |  |  |  |
| 67 | W |  |  |  |  |  |  |  |  |  |  |  |  |  |  |  |
| 68 | M |  |  |  |  |  |  |  |  |  |  |  |  |  |  |  |
| 68 | W |  |  |  |  |  |  |  |  |  |  |  |  |  |  |  |
| 69 | M | 0.106 | 0.071 | 0.141 |  |  |  |  |  |  |  |  |  |  |  |  |
| 69 | W | 0.131 | 0.094 | 0.168 |  |  |  |  |  |  |  |  |  |  |  |  |
| 70 | M |  |  |  | 0.128 | 0.095 | 0.162 |  |  |  |  |  |  |  |  |  |
| 70 | W |  |  |  | 0.189 | 0.150 | 0.228 |  |  |  |  |  |  |  |  |  |
| 71 | M | 0.118 | 0.081 | 0.155 |  |  |  |  |  |  |  |  |  |  |  |  |
| 71 | W | 0.138 | 0.100 | 0.177 |  |  |  |  |  |  |  |  |  |  |  |  |
| 72 | M |  |  |  |  |  |  |  |  |  |  |  |  |  |  |  |
| 72 | W |  |  |  |  |  |  |  |  |  |  |  |  |  |  |  |
| 73 | M | 0.134 | 0.093 | 0.175 |  |  |  |  |  |  |  |  |  |  |  |  |
| 73 | W | 0.151 | 0.110 | 0.193 |  |  |  |  |  |  |  |  |  |  |  |  |
| 74 | M |  |  |  | 0.153 | 0.122 | 0.185 |  |  |  |  |  |  |  |  |  |
| 74 | W |  |  |  | 0.199 | 0.164 | 0.234 |  |  |  |  |  |  |  |  |  |
| 75 | M | 0.155 | 0.107 | 0.203 |  |  |  | 0.163 | 0.119 | 0.206 |  |  |  |  |  |  |
| 75 | W | 0.169 | 0.121 | 0.217 |  |  |  | 0.252 | 0.201 | 0.303 |  |  |  |  |  |  |
| 76 | M |  |  |  | 0.177 | 0.142 | 0.212 |  |  |  |  |  |  |  |  |  |
| 76 | W |  |  |  | 0.217 | 0.180 | 0.253 |  |  |  |  |  |  |  |  |  |
| 77 | M |  |  |  |  |  |  |  |  |  |  |  |  |  |  |  |
| 77 | W |  |  |  |  |  |  |  |  |  |  |  |  |  |  |  |
| 78 | M |  |  |  | 0.205 | 0.166 | 0.244 |  |  |  |  |  |  |  |  |  |
| 78 | W |  |  |  | 0.239 | 0.201 | 0.277 |  |  |  |  |  |  |  |  |  |
| 79 | M |  |  |  |  |  |  | 0.219 | 0.175 | 0.262 |  |  |  |  |  |  |
| 79 | W |  |  |  |  |  |  | 0.292 | 0.246 | 0.338 |  |  |  |  |  |  |
| 80 | M |  |  |  | 0.236 | 0.189 | 0.283 |  |  |  | 0.208 | 0.136 | 0.281 |  |  |  |
| 80 | W |  |  |  | 0.264 | 0.219 | 0.309 |  |  |  | 0.334 | 0.267 | 0.400 |  |  |  |
| 81 | M |  |  |  |  |  |  | 0.257 | 0.210 | 0.304 |  |  |  |  |  |  |
| 81 | W |  |  |  |  |  |  | 0.321 | 0.275 | 0.368 |  |  |  |  |  |  |
| 82 | M |  |  |  |  |  |  |  |  |  |  |  |  |  |  |  |
| 82 | W |  |  |  |  |  |  |  |  |  |  |  |  |  |  |  |
| 83 | M |  |  |  |  |  |  | 0.298 | 0.246 | 0.349 |  |  |  |  |  |  |
| 83 | W |  |  |  |  |  |  | 0.354 | 0.305 | 0.402 |  |  |  |  |  |  |
| 84 | M |  |  |  |  |  |  |  |  |  | 0.304 | 0.233 | 0.375 | 0.269 | 0.127 | 0.410 |
| 84 | W |  |  |  |  |  |  |  |  |  | 0.408 | 0.353 | 0.463 |  |  |  |
| 85 | M |  |  |  |  |  |  | 0.338 | 0.276 | 0.400 |  |  |  |  |  |  |
| 85 | W |  |  |  |  |  |  | 0.386 | 0.329 | 0.444 |  |  |  | 0.433 | 0.316 | 0.550 |
| 86 | M |  |  |  |  |  |  |  |  |  | 0.359 | 0.284 | 0.433 |  |  |  |
| 86 | W |  |  |  |  |  |  |  |  |  | 0.450 | 0.392 | 0.507 |  |  |  |
| 87 | M |  |  |  |  |  |  |  |  |  |  |  |  |  |  |  |
| 87 | W |  |  |  |  |  |  |  |  |  |  |  |  |  |  |  |
| 88 | M |  |  |  |  |  |  |  |  |  | 0.412 | 0.332 | 0.492 | 0.409 | 0.277 | 0.541 |
| 88 | W |  |  |  |  |  |  |  |  |  | 0.491 | 0.428 | 0.555 |  |  |  |
| 89 | M |  |  |  |  |  |  |  |  |  |  |  |  |  |  |  |
| 89 | W |  |  |  |  |  |  |  |  |  |  |  |  | 0.540 | 0.450 | 0.630 |
| 90 | M |  |  |  |  |  |  |  |  |  | 0.465 | 0.371 | 0.558 | 0.478 | 0.350 | 0.607 |
| 90 | W |  |  |  |  |  |  |  |  |  | 0.533 | 0.457 | 0.609 | 0.590 | 0.503 | 0.677 |
| 91 | M |  |  |  |  |  |  |  |  |  |  |  |  |  |  |  |
| 91 | W |  |  |  |  |  |  |  |  |  |  |  |  |  |  |  |
| 92 | M |  |  |  |  |  |  |  |  |  |  |  |  | 0.543 | 0.415 | 0.670 |
| 92 | W |  |  |  |  |  |  |  |  |  |  |  |  | 0.636 | 0.548 | 0.723 |
| 93 | M |  |  |  |  |  |  |  |  |  |  |  |  |  |  |  |
| 93 | W |  |  |  |  |  |  |  |  |  |  |  |  |  |  |  |
| 94 | M |  |  |  |  |  |  |  |  |  |  |  |  | 0.602 | 0.473 | 0.731 |
| 94 | W |  |  |  |  |  |  |  |  |  |  |  |  |  |  |  |
| 95 | M |  |  |  |  |  |  |  |  |  |  |  |  |  |  |  |
| 95 | W |  |  |  |  |  |  |  |  |  |  |  |  | 0.677 | 0.586 | 0.768 |

**Supplementary table 4.** Predicted probabilities of **ADL** limitations by age and sex (**model 1**). **Northern Europe**, data for figure 1. (PP, predicted probability. LCI, lower confidence interval. UCI, upper confidence interval).

|  |  | Cohort 1940-1944 | | | Cohort 1935-1939 | | | Cohort 1930-1934 | | | Cohort 1925-1929 | | | Cohort 1920-1924 | | |
| --- | --- | --- | --- | --- | --- | --- | --- | --- | --- | --- | --- | --- | --- | --- | --- | --- |
| Age | Sex | PP | LCI | UCI | PP | LCI | UCI | PP | LCI | UCI | PP | LCI | UCI | PP | LCI | UCI |
| 62 | M | 0.054 | 0.038 | 0.070 |  |  |  |  |  |  |  |  |  |  |  |  |
| 62 | W | 0.057 | 0.040 | 0.073 |  |  |  |  |  |  |  |  |  |  |  |  |
| … |  |  |  |  |  |  |  |  |  |  |  |  |  |  |  |  |
| 65 | M | 0.059 | 0.045 | 0.073 |  |  |  |  |  |  |  |  |  |  |  |  |
| 65 | W | 0.058 | 0.043 | 0.072 |  |  |  |  |  |  |  |  |  |  |  |  |
| 66 | M |  |  |  |  |  |  |  |  |  |  |  |  |  |  |  |
| 66 | W |  |  |  |  |  |  |  |  |  |  |  |  |  |  |  |
| 67 | M |  |  |  | 0.056 | 0.043 | 0.068 |  |  |  |  |  |  |  |  |  |
| 67 | W |  |  |  | 0.061 | 0.047 | 0.075 |  |  |  |  |  |  |  |  |  |
| 68 | M |  |  |  |  |  |  |  |  |  |  |  |  |  |  |  |
| 68 | W |  |  |  |  |  |  |  |  |  |  |  |  |  |  |  |
| 69 | M | 0.077 | 0.060 | 0.094 |  |  |  |  |  |  |  |  |  |  |  |  |
| 69 | W | 0.067 | 0.052 | 0.082 |  |  |  |  |  |  |  |  |  |  |  |  |
| 70 | M |  |  |  | 0.068 | 0.055 | 0.080 |  |  |  |  |  |  |  |  |  |
| 70 | W |  |  |  | 0.070 | 0.057 | 0.083 |  |  |  |  |  |  |  |  |  |
| 71 | M | 0.089 | 0.070 | 0.107 |  |  |  |  |  |  |  |  |  |  |  |  |
| 71 | W | 0.074 | 0.059 | 0.090 |  |  |  |  |  |  |  |  |  |  |  |  |
| 72 | M |  |  |  |  |  |  | 0.066 | 0.050 | 0.083 |  |  |  |  |  |  |
| 72 | W |  |  |  |  |  |  | 0.077 | 0.060 | 0.095 |  |  |  |  |  |  |
| 73 | M | 0.101 | 0.080 | 0.122 |  |  |  |  |  |  |  |  |  |  |  |  |
| 73 | W | 0.083 | 0.065 | 0.100 |  |  |  |  |  |  |  |  |  |  |  |  |
| 74 | M |  |  |  | 0.104 | 0.088 | 0.120 |  |  |  |  |  |  |  |  |  |
| 74 | W |  |  |  | 0.096 | 0.081 | 0.111 |  |  |  |  |  |  |  |  |  |
| 75 | M | 0.114 | 0.089 | 0.139 |  |  |  | 0.089 | 0.071 | 0.106 |  |  |  |  |  |  |
| 75 | W | 0.091 | 0.071 | 0.111 |  |  |  | 0.096 | 0.079 | 0.114 |  |  |  |  |  |  |
| 76 | M |  |  |  | 0.126 | 0.108 | 0.144 |  |  |  |  |  |  |  |  |  |
| 76 | W |  |  |  | 0.112 | 0.096 | 0.128 |  |  |  |  |  |  |  |  |  |
| 77 | M |  |  |  |  |  |  |  |  |  | 0.090 | 0.066 | 0.115 |  |  |  |
| 77 | W |  |  |  |  |  |  |  |  |  | 0.110 | 0.086 | 0.134 |  |  |  |
| 78 | M |  |  |  | 0.149 | 0.128 | 0.170 |  |  |  |  |  |  |  |  |  |
| 78 | W |  |  |  | 0.129 | 0.111 | 0.147 |  |  |  |  |  |  |  |  |  |
| 79 | M |  |  |  |  |  |  | 0.153 | 0.130 | 0.176 |  |  |  |  |  |  |
| 79 | W |  |  |  |  |  |  | 0.149 | 0.127 | 0.171 |  |  |  |  |  |  |
| 80 | M |  |  |  | 0.174 | 0.146 | 0.201 |  |  |  | 0.130 | 0.103 | 0.156 |  |  |  |
| 80 | W |  |  |  | 0.146 | 0.124 | 0.169 |  |  |  | 0.147 | 0.122 | 0.171 |  |  |  |
| 81 | M |  |  |  |  |  |  | 0.191 | 0.165 | 0.218 |  |  |  |  |  |  |
| 81 | W |  |  |  |  |  |  | 0.179 | 0.155 | 0.203 |  |  |  |  |  |  |
| 82 | M |  |  |  |  |  |  |  |  |  |  |  |  | 0.138 | 0.089 | 0.187 |
| 82 | W |  |  |  |  |  |  |  |  |  |  |  |  | 0.172 | 0.125 | 0.219 |
| 83 | M |  |  |  |  |  |  | 0.232 | 0.201 | 0.262 |  |  |  |  |  |  |
| 83 | W |  |  |  |  |  |  | 0.211 | 0.184 | 0.238 |  |  |  |  |  |  |
| 84 | M |  |  |  |  |  |  |  |  |  | 0.237 | 0.203 | 0.271 |  |  |  |
| 84 | W |  |  |  |  |  |  |  |  |  | 0.239 | 0.209 | 0.270 |  |  |  |
| 85 | M |  |  |  |  |  |  | 0.271 | 0.234 | 0.308 |  |  |  | 0.204 | 0.149 | 0.259 |
| 85 | W |  |  |  |  |  |  | 0.242 | 0.208 | 0.276 |  |  |  | 0.235 | 0.185 | 0.284 |
| 86 | M |  |  |  |  |  |  |  |  |  | 0.297 | 0.259 | 0.336 |  |  |  |
| 86 | W |  |  |  |  |  |  |  |  |  | 0.290 | 0.256 | 0.324 |  |  |  |
| 87 | M |  |  |  |  |  |  |  |  |  |  |  |  |  |  |  |
| 87 | W |  |  |  |  |  |  |  |  |  |  |  |  |  |  |  |
| 88 | M |  |  |  |  |  |  |  |  |  | 0.355 | 0.312 | 0.398 |  |  |  |
| 88 | W |  |  |  |  |  |  |  |  |  | 0.338 | 0.300 | 0.376 |  |  |  |
| 89 | M |  |  |  |  |  |  |  |  |  |  |  |  | 0.367 | 0.301 | 0.434 |
| 89 | W |  |  |  |  |  |  |  |  |  |  |  |  | 0.380 | 0.321 | 0.439 |
| 90 | M |  |  |  |  |  |  |  |  |  | 0.407 | 0.354 | 0.460 |  |  |  |
| 90 | W |  |  |  |  |  |  |  |  |  | 0.381 | 0.333 | 0.429 |  |  |  |
| 91 | M |  |  |  |  |  |  |  |  |  |  |  |  | 0.448 | 0.378 | 0.519 |
| 91 | W |  |  |  |  |  |  |  |  |  |  |  |  | 0.450 | 0.387 | 0.513 |
| 92 | M |  |  |  |  |  |  |  |  |  |  |  |  |  |  |  |
| 92 | W |  |  |  |  |  |  |  |  |  |  |  |  |  |  |  |
| 93 | M |  |  |  |  |  |  |  |  |  |  |  |  | 0.522 | 0.447 | 0.597 |
| 93 | W |  |  |  |  |  |  |  |  |  |  |  |  | 0.512 | 0.444 | 0.581 |
| 94 | M |  |  |  |  |  |  |  |  |  |  |  |  |  |  |  |
| 94 | W |  |  |  |  |  |  |  |  |  |  |  |  | 0.565 | 0.491 | 0.640 |
| 95 | M |  |  |  |  |  |  |  |  |  |  |  |  | 0.584 | 0.505 | 0.663 |

**Supplementary table 5.** Predicted probabilities of **ADL** limitations by age and sex (**model 1**)**. Southern Europe**, data for figure 1. (PP, predicted probability. LCI, lower confidence interval. UCI, upper confidence interval).

|  |  | Cohort 1940-1944 | | | Cohort 1935-1939 | | | Cohort 1930-1934 | | | Cohort 1925-1929 | | | Cohort 1920-1924 | | |
| --- | --- | --- | --- | --- | --- | --- | --- | --- | --- | --- | --- | --- | --- | --- | --- | --- |
| Age | Sex | PP | LCI | UCI | PP | LCI | UCI | PP | LCI | UCI | PP | LCI | UCI | PP | LCI | UCI |
| 62 | M | 0.040 | 0.027 | 0.052 |  |  |  |  |  |  |  |  |  |  |  |  |
| 62 | W | 0.065 | 0.049 | 0.081 |  |  |  |  |  |  |  |  |  |  |  |  |
| … |  |  |  |  |  |  |  |  |  |  |  |  |  |  |  |  |
| 65 | M | 0.042 | 0.031 | 0.053 |  |  |  |  |  |  |  |  |  |  |  |  |
| 65 | W | 0.069 | 0.055 | 0.083 |  |  |  |  |  |  |  |  |  |  |  |  |
| 66 | M |  |  |  |  |  |  |  |  |  |  |  |  |  |  |  |
| 66 | W |  |  |  |  |  |  |  |  |  |  |  |  |  |  |  |
| 67 | M |  |  |  | 0.053 | 0.041 | 0.064 |  |  |  |  |  |  |  |  |  |
| 67 | W |  |  |  | 0.085 | 0.070 | 0.100 |  |  |  |  |  |  |  |  |  |
| 68 | M |  |  |  |  |  |  |  |  |  |  |  |  |  |  |  |
| 68 | W |  |  |  |  |  |  |  |  |  |  |  |  |  |  |  |
| 69 | M | 0.054 | 0.041 | 0.066 |  |  |  |  |  |  |  |  |  |  |  |  |
| 69 | W | 0.088 | 0.073 | 0.103 |  |  |  |  |  |  |  |  |  |  |  |  |
| 70 | M |  |  |  | 0.062 | 0.052 | 0.072 |  |  |  |  |  |  |  |  |  |
| 70 | W |  |  |  | 0.100 | 0.087 | 0.114 |  |  |  |  |  |  |  |  |  |
| 71 | M | 0.063 | 0.049 | 0.077 |  |  |  |  |  |  |  |  |  |  |  |  |
| 71 | W | 0.102 | 0.085 | 0.119 |  |  |  |  |  |  |  |  |  |  |  |  |
| 72 | M |  |  |  |  |  |  | 0.079 | 0.063 | 0.095 |  |  |  |  |  |  |
| 72 | W |  |  |  |  |  |  | 0.125 | 0.106 | 0.145 |  |  |  |  |  |  |
| 73 | M | 0.075 | 0.059 | 0.091 |  |  |  |  |  |  |  |  |  |  |  |  |
| 73 | W | 0.119 | 0.100 | 0.138 |  |  |  |  |  |  |  |  |  |  |  |  |
| 74 | M |  |  |  | 0.093 | 0.080 | 0.106 |  |  |  |  |  |  |  |  |  |
| 74 | W |  |  |  | 0.146 | 0.130 | 0.163 |  |  |  |  |  |  |  |  |  |
| 75 | M | 0.089 | 0.070 | 0.109 |  |  |  | 0.101 | 0.086 | 0.117 |  |  |  |  |  |  |
| 75 | W | 0.137 | 0.114 | 0.161 |  |  |  | 0.158 | 0.139 | 0.178 |  |  |  |  |  |  |
| 76 | M |  |  |  | 0.115 | 0.100 | 0.130 |  |  |  |  |  |  |  |  |  |
| 76 | W |  |  |  | 0.176 | 0.158 | 0.194 |  |  |  |  |  |  |  |  |  |
| 77 | M |  |  |  |  |  |  |  |  |  | 0.130 | 0.103 | 0.157 |  |  |  |
| 77 | W |  |  |  |  |  |  |  |  |  | 0.197 | 0.169 | 0.226 |  |  |  |
| 78 | M |  |  |  | 0.141 | 0.123 | 0.159 |  |  |  |  |  |  |  |  |  |
| 78 | W |  |  |  | 0.209 | 0.189 | 0.230 |  |  |  |  |  |  |  |  |  |
| 79 | M |  |  |  |  |  |  | 0.168 | 0.147 | 0.189 |  |  |  |  |  |  |
| 79 | W |  |  |  |  |  |  | 0.247 | 0.222 | 0.272 |  |  |  |  |  |  |
| 80 | M |  |  |  | 0.169 | 0.145 | 0.193 |  |  |  | 0.176 | 0.147 | 0.204 |  |  |  |
| 80 | W |  |  |  | 0.245 | 0.218 | 0.272 |  |  |  | 0.258 | 0.229 | 0.287 |  |  |  |
| 81 | M |  |  |  |  |  |  | 0.211 | 0.187 | 0.234 |  |  |  |  |  |  |
| 81 | W |  |  |  |  |  |  | 0.300 | 0.274 | 0.326 |  |  |  |  |  |  |
| 82 | M |  |  |  |  |  |  |  |  |  |  |  |  | 0.223 | 0.165 | 0.281 |
| 82 | W |  |  |  |  |  |  |  |  |  |  |  |  | 0.315 | 0.256 | 0.374 |
| 83 | M |  |  |  |  |  |  | 0.258 | 0.231 | 0.286 |  |  |  |  |  |  |
| 83 | W |  |  |  |  |  |  | 0.354 | 0.326 | 0.382 |  |  |  |  |  |  |
| 84 | M |  |  |  |  |  |  |  |  |  | 0.297 | 0.262 | 0.332 |  |  |  |
| 84 | W |  |  |  |  |  |  |  |  |  | 0.404 | 0.369 | 0.439 |  |  |  |
| 85 | M |  |  |  |  |  |  | 0.307 | 0.273 | 0.342 |  |  |  | 0.302 | 0.240 | 0.365 |
| 85 | W |  |  |  |  |  |  | 0.407 | 0.372 | 0.441 |  |  |  | 0.411 | 0.350 | 0.472 |
| 86 | M |  |  |  |  |  |  |  |  |  | 0.367 | 0.329 | 0.405 |  |  |  |
| 86 | W |  |  |  |  |  |  |  |  |  | 0.479 | 0.443 | 0.515 |  |  |  |
| 87 | M |  |  |  |  |  |  |  |  |  |  |  |  |  |  |  |
| 87 | W |  |  |  |  |  |  |  |  |  |  |  |  |  |  |  |
| 88 | M |  |  |  |  |  |  |  |  |  | 0.436 | 0.393 | 0.478 |  |  |  |
| 88 | W |  |  |  |  |  |  |  |  |  | 0.549 | 0.511 | 0.587 |  |  |  |
| 89 | M |  |  |  |  |  |  |  |  |  |  |  |  | 0.487 | 0.420 | 0.555 |
| 89 | W |  |  |  |  |  |  |  |  |  |  |  |  | 0.605 | 0.546 | 0.664 |
| 90 | M |  |  |  |  |  |  |  |  |  | 0.502 | 0.451 | 0.553 |  |  |  |
| 90 | W |  |  |  |  |  |  |  |  |  | 0.612 | 0.569 | 0.655 | 0.685 | 0.630 | 0.740 |
| 91 | M |  |  |  |  |  |  |  |  |  |  |  |  | 0.575 | 0.509 | 0.641 |
| 91 | W |  |  |  |  |  |  |  |  |  |  |  |  |  |  |  |
| 92 | M |  |  |  |  |  |  |  |  |  |  |  |  | 0.652 | 0.588 | 0.715 |
| 92 | W |  |  |  |  |  |  |  |  |  |  |  |  |  |  |  |
| 93 | M |  |  |  |  |  |  |  |  |  |  |  |  |  |  |  |
| 93 | W |  |  |  |  |  |  |  |  |  |  |  |  | 0.751 | 0.699 | 0.803 |
| 94 | M |  |  |  |  |  |  |  |  |  |  |  |  | 0.715 | 0.651 | 0.779 |
| 94 | W |  |  |  |  |  |  |  |  |  |  |  |  | 0.803 | 0.753 | 0.853 |

**Supplementary table 6.** Predicted probabilities of **ADL** limitations by age and sex (**model 1**). **Western Europe**, data for figure 1. (PP, predicted probability. LCI, lower confidence interval. UCI, upper confidence interval).

|  |  | Cohort 1940-1944 | | | Cohort 1935-1939 | | | Cohort 1930-1934 | | | Cohort 1925-1929 | | | Cohort 1920-1924 | | |
| --- | --- | --- | --- | --- | --- | --- | --- | --- | --- | --- | --- | --- | --- | --- | --- | --- |
| Age | Sex | PP | LCI | UCI | PP | LCI | UCI | PP | LCI | UCI | PP | LCI | UCI | PP | LCI | UCI |
| 62 | M | 0.057 | 0.042 | 0.073 |  |  |  |  |  |  |  |  |  |  |  |  |
| 62 | W | 0.067 | 0.052 | 0.083 |  |  |  |  |  |  |  |  |  |  |  |  |
| … |  |  |  |  |  |  |  |  |  |  |  |  |  |  |  |  |
| 65 | M | 0.066 | 0.051 | 0.081 |  |  |  |  |  |  |  |  |  |  |  |  |
| 65 | W | 0.075 | 0.060 | 0.089 |  |  |  |  |  |  |  |  |  |  |  |  |
| 66 | M |  |  |  |  |  |  |  |  |  |  |  |  |  |  |  |
| 66 | W |  |  |  |  |  |  |  |  |  |  |  |  |  |  |  |
| 67 | M |  |  |  | 0.063 | 0.052 | 0.075 |  |  |  |  |  |  |  |  |  |
| 67 | W |  |  |  | 0.077 | 0.064 | 0.089 |  |  |  |  |  |  |  |  |  |
| 68 | M |  |  |  |  |  |  |  |  |  |  |  |  |  |  |  |
| 68 | W |  |  |  |  |  |  |  |  |  |  |  |  |  |  |  |
| 69 | M | 0.093 | 0.076 | 0.110 |  |  |  |  |  |  |  |  |  |  |  |  |
| 69 | W | 0.097 | 0.081 | 0.114 |  |  |  |  |  |  |  |  |  |  |  |  |
| 70 | M |  |  |  | 0.080 | 0.069 | 0.092 |  |  |  |  |  |  |  |  |  |
| 70 | W |  |  |  | 0.093 | 0.081 | 0.105 |  |  |  |  |  |  |  |  |  |
| 71 | M | 0.110 | 0.091 | 0.129 |  |  |  |  |  |  |  |  |  |  |  |  |
| 71 | W | 0.112 | 0.094 | 0.129 |  |  |  |  |  |  |  |  |  |  |  |  |
| 72 | M |  |  |  |  |  |  | 0.082 | 0.068 | 0.096 |  |  |  |  |  |  |
| 72 | W |  |  |  |  |  |  | 0.101 | 0.085 | 0.117 |  |  |  |  |  |  |
| 73 | M | 0.129 | 0.107 | 0.151 |  |  |  |  |  |  |  |  |  |  |  |  |
| 73 | W | 0.127 | 0.107 | 0.147 |  |  |  |  |  |  |  |  |  |  |  |  |
| 74 | M |  |  |  | 0.128 | 0.114 | 0.143 |  |  |  |  |  |  |  |  |  |
| 74 | W |  |  |  | 0.137 | 0.122 | 0.151 |  |  |  |  |  |  |  |  |  |
| 75 | M | 0.148 | 0.121 | 0.176 |  |  |  | 0.111 | 0.097 | 0.126 |  |  |  |  |  |  |
| 75 | W | 0.143 | 0.118 | 0.168 |  |  |  | 0.130 | 0.114 | 0.147 |  |  |  |  |  |  |
| 76 | M |  |  |  | 0.158 | 0.141 | 0.176 |  |  |  |  |  |  |  |  |  |
| 76 | W |  |  |  | 0.163 | 0.146 | 0.179 |  |  |  |  |  |  |  |  |  |
| 77 | M |  |  |  |  |  |  |  |  |  | 0.120 | 0.099 | 0.142 |  |  |  |
| 77 | W |  |  |  |  |  |  |  |  |  | 0.149 | 0.127 | 0.171 |  |  |  |
| 78 | M |  |  |  | 0.190 | 0.168 | 0.212 |  |  |  |  |  |  |  |  |  |
| 78 | W |  |  |  | 0.191 | 0.171 | 0.211 |  |  |  |  |  |  |  |  |  |
| 79 | M |  |  |  |  |  |  | 0.192 | 0.172 | 0.212 |  |  |  |  |  |  |
| 79 | W |  |  |  |  |  |  | 0.206 | 0.185 | 0.227 |  |  |  |  |  |  |
| 80 | M |  |  |  | 0.224 | 0.194 | 0.253 |  |  |  | 0.170 | 0.147 | 0.193 |  |  |  |
| 80 | W |  |  |  | 0.220 | 0.193 | 0.247 |  |  |  | 0.198 | 0.177 | 0.220 |  |  |  |
| 81 | M |  |  |  |  |  |  | 0.240 | 0.216 | 0.264 |  |  |  |  |  |  |
| 81 | W |  |  |  |  |  |  | 0.250 | 0.227 | 0.274 |  |  |  |  |  |  |
| 82 | M |  |  |  |  |  |  |  |  |  |  |  |  | 0.192 | 0.147 | 0.236 |
| 82 | W |  |  |  |  |  |  |  |  |  |  |  |  | 0.233 | 0.193 | 0.273 |
| 83 | M |  |  |  |  |  |  | 0.290 | 0.261 | 0.319 |  |  |  |  |  |  |
| 83 | W |  |  |  |  |  |  | 0.295 | 0.268 | 0.322 |  |  |  |  |  |  |
| 84 | M |  |  |  |  |  |  |  |  |  | 0.297 | 0.265 | 0.329 |  |  |  |
| 84 | W |  |  |  |  |  |  |  |  |  | 0.320 | 0.291 | 0.348 |  |  |  |
| 85 | M |  |  |  |  |  |  | 0.338 | 0.302 | 0.374 |  |  |  | 0.269 | 0.220 | 0.319 |
| 85 | W |  |  |  |  |  |  | 0.337 | 0.304 | 0.371 |  |  |  | 0.311 | 0.268 | 0.354 |
| 86 | M |  |  |  |  |  |  |  |  |  | 0.366 | 0.330 | 0.403 |  |  |  |
| 86 | W |  |  |  |  |  |  |  |  |  | 0.383 | 0.351 | 0.415 |  |  |  |
| 87 | M |  |  |  |  |  |  |  |  |  |  |  |  |  |  |  |
| 87 | W |  |  |  |  |  |  |  |  |  |  |  |  |  |  |  |
| 88 | M |  |  |  |  |  |  |  |  |  | 0.433 | 0.390 | 0.476 |  |  |  |
| 88 | W |  |  |  |  |  |  |  |  |  | 0.443 | 0.406 | 0.481 |  |  |  |
| 89 | M |  |  |  |  |  |  |  |  |  | 0.495 | 0.443 | 0.547 | 0.452 | 0.392 | 0.513 |
| 89 | W |  |  |  |  |  |  |  |  |  |  |  |  | 0.483 | 0.433 | 0.534 |
| 90 | M |  |  |  |  |  |  |  |  |  |  |  |  | 0.539 | 0.476 | 0.602 |
| 90 | W |  |  |  |  |  |  |  |  |  | 0.499 | 0.453 | 0.545 | 0.562 | 0.509 | 0.615 |
| 91 | M |  |  |  |  |  |  |  |  |  |  |  |  |  |  |  |
| 91 | W |  |  |  |  |  |  |  |  |  |  |  |  |  |  |  |
| 92 | M |  |  |  |  |  |  |  |  |  |  |  |  | 0.615 | 0.551 | 0.678 |
| 92 | W |  |  |  |  |  |  |  |  |  |  |  |  | 0.629 | 0.575 | 0.683 |
| 93 | M |  |  |  |  |  |  |  |  |  |  |  |  |  |  |  |
| 93 | W |  |  |  |  |  |  |  |  |  |  |  |  |  |  |  |
| 94 | M |  |  |  |  |  |  |  |  |  |  |  |  | 0.676 | 0.612 | 0.740 |
| 94 | W |  |  |  |  |  |  |  |  |  |  |  |  | 0.683 | 0.627 | 0.739 |

**Supplementary table 7.** Predicted probabilities of **ADL** limitations by age and sex adjusted for income and education (**model 2**). **Eastern Europe**, data for figure 1. (PP, predicted probability. LCI, lower confidence interval. UCI, upper confidence interval).

|  |  | Cohort 1940-1944 | | | Cohort 1935-1939 | | | Cohort 1930-1934 | | | Cohort 1925-1929 | | | Cohort 1920-1924 | | |
| --- | --- | --- | --- | --- | --- | --- | --- | --- | --- | --- | --- | --- | --- | --- | --- | --- |
| Age | Sex | PP | LCI | UCI | PP | LCI | UCI | PP | LCI | UCI | PP | LCI | UCI | PP | LCI | UCI |
| … | … |  |  |  |  |  |  |  |  |  |  |  |  |  |  |  |
| 65 | M | 0.114 | 0.071 | 0.158 |  |  |  |  |  |  |  |  |  |  |  |  |
| 65 | W | 0.144 | 0.098 | 0.191 |  |  |  |  |  |  |  |  |  |  |  |  |
| 66 | M |  |  |  |  |  |  |  |  |  |  |  |  |  |  |  |
| 66 | W |  |  |  |  |  |  |  |  |  |  |  |  |  |  |  |
| 67 | M |  |  |  |  |  |  |  |  |  |  |  |  |  |  |  |
| 67 | W |  |  |  |  |  |  |  |  |  |  |  |  |  |  |  |
| 68 | M |  |  |  |  |  |  |  |  |  |  |  |  |  |  |  |
| 68 | W |  |  |  |  |  |  |  |  |  |  |  |  |  |  |  |
| 69 | M | 0.117 | 0.080 | 0.154 |  |  |  |  |  |  |  |  |  |  |  |  |
| 69 | W | 0.134 | 0.096 | 0.171 |  |  |  |  |  |  |  |  |  |  |  |  |
| 70 | M |  |  |  | 0.136 | 0.101 | 0.171 |  |  |  |  |  |  |  |  |  |
| 70 | W |  |  |  | 0.182 | 0.144 | 0.220 |  |  |  |  |  |  |  |  |  |
| 71 | M | 0.128 | 0.089 | 0.168 |  |  |  |  |  |  |  |  |  |  |  |  |
| 71 | W | 0.141 | 0.102 | 0.180 |  |  |  |  |  |  |  |  |  |  |  |  |
| 72 | M |  |  |  |  |  |  |  |  |  |  |  |  |  |  |  |
| 72 | W |  |  |  |  |  |  |  |  |  |  |  |  |  |  |  |
| 73 | M | 0.145 | 0.102 | 0.187 |  |  |  |  |  |  |  |  |  |  |  |  |
| 73 | W | 0.153 | 0.111 | 0.195 |  |  |  |  |  |  |  |  |  |  |  |  |
| 74 | M |  |  |  | 0.162 | 0.130 | 0.195 |  |  |  |  |  |  |  |  |  |
| 74 | W |  |  |  | 0.194 | 0.159 | 0.228 |  |  |  |  |  |  |  |  |  |
| 75 | M | 0.165 | 0.115 | 0.215 |  |  |  | 0.167 | 0.123 | 0.211 |  |  |  |  |  |  |
| 75 | W | 0.169 | 0.121 | 0.218 |  |  |  | 0.234 | 0.185 | 0.282 |  |  |  |  |  |  |
| 76 | M |  |  |  | 0.185 | 0.150 | 0.221 |  |  |  |  |  |  |  |  |  |
| 76 | W |  |  |  | 0.211 | 0.175 | 0.247 |  |  |  |  |  |  |  |  |  |
| 77 | M |  |  |  |  |  |  |  |  |  |  |  |  |  |  |  |
| 77 | W |  |  |  |  |  |  |  |  |  |  |  |  |  |  |  |
| 78 | M |  |  |  | 0.213 | 0.174 | 0.252 |  |  |  |  |  |  |  |  |  |
| 78 | W |  |  |  | 0.233 | 0.195 | 0.271 |  |  |  |  |  |  |  |  |  |
| 79 | M |  |  |  |  |  |  | 0.224 | 0.181 | 0.268 |  |  |  |  |  |  |
| 79 | W |  |  |  |  |  |  | 0.276 | 0.231 | 0.321 |  |  |  |  |  |  |
| 80 | M |  |  |  | 0.243 | 0.195 | 0.291 |  |  |  | 0.210 | 0.139 | 0.280 |  |  |  |
| 80 | W |  |  |  | 0.258 | 0.213 | 0.303 |  |  |  | 0.302 | 0.238 | 0.366 |  |  |  |
| 81 | M |  |  |  |  |  |  | 0.262 | 0.215 | 0.309 |  |  |  |  |  |  |
| 81 | W |  |  |  |  |  |  | 0.306 | 0.260 | 0.352 |  |  |  |  |  |  |
| 82 | M |  |  |  |  |  |  |  |  |  |  |  |  |  |  |  |
| 82 | W |  |  |  |  |  |  |  |  |  |  |  |  |  |  |  |
| 83 | M |  |  |  |  |  |  | 0.303 | 0.251 | 0.355 |  |  |  |  |  |  |
| 83 | W |  |  |  |  |  |  | 0.339 | 0.291 | 0.387 |  |  |  |  |  |  |
| 84 | M |  |  |  |  |  |  |  |  |  | 0.306 | 0.237 | 0.375 | 0.267 | 0.132 | 0.402 |
| 84 | W |  |  |  |  |  |  |  |  |  | 0.380 | 0.325 | 0.436 |  |  |  |
| 85 | M |  |  |  |  |  |  | 0.344 | 0.281 | 0.407 |  |  |  |  |  |  |
| 85 | W |  |  |  |  |  |  | 0.372 | 0.315 | 0.430 |  |  |  | 0.389 | 0.276 | 0.502 |
| 86 | M |  |  |  |  |  |  |  |  |  | 0.361 | 0.288 | 0.434 |  |  |  |
| 86 | W |  |  |  |  |  |  |  |  |  | 0.424 | 0.366 | 0.482 |  |  |  |
| 87 | M |  |  |  |  |  |  |  |  |  |  |  |  |  |  |  |
| 87 | W |  |  |  |  |  |  |  |  |  |  |  |  |  |  |  |
| 88 | M |  |  |  |  |  |  |  |  |  | 0.415 | 0.335 | 0.494 | 0.408 | 0.280 | 0.535 |
| 88 | W |  |  |  |  |  |  |  |  |  | 0.468 | 0.404 | 0.531 |  |  |  |
| 89 | M |  |  |  |  |  |  |  |  |  |  |  |  |  |  |  |
| 89 | W |  |  |  |  |  |  |  |  |  |  |  |  | 0.504 | 0.414 | 0.594 |
| 90 | M |  |  |  |  |  |  |  |  |  | 0.467 | 0.375 | 0.559 | 0.478 | 0.353 | 0.603 |
| 90 | W |  |  |  |  |  |  |  |  |  | 0.511 | 0.435 | 0.587 | 0.558 | 0.470 | 0.647 |
| 91 | M |  |  |  |  |  |  |  |  |  |  |  |  |  |  |  |
| 91 | W |  |  |  |  |  |  |  |  |  |  |  |  |  |  |  |
| 92 | M |  |  |  |  |  |  |  |  |  |  |  |  | 0.543 | 0.418 | 0.668 |
| 92 | W |  |  |  |  |  |  |  |  |  |  |  |  | 0.608 | 0.517 | 0.698 |
| 93 | M |  |  |  |  |  |  |  |  |  |  |  |  |  |  |  |
| 93 | W |  |  |  |  |  |  |  |  |  |  |  |  |  |  |  |
| 94 | M |  |  |  |  |  |  |  |  |  |  |  |  | 0.601 | 0.474 | 0.729 |
| 94 | W |  |  |  |  |  |  |  |  |  |  |  |  |  |  |  |
| 95 | M |  |  |  |  |  |  |  |  |  |  |  |  |  |  |  |
| 95 | W |  |  |  |  |  |  |  |  |  |  |  |  | 0.653 | 0.557 | 0.748 |

**Supplementary table 8.** Predicted probabilities of **ADL** limitations by age and sex adjusted for income and education (**model 2**). **Northern Europe**, data for figure 1. (PP, predicted probability. LCI, lower confidence interval. UCI, upper confidence interval).

|  |  | Cohort 1940-1944 | | | Cohort 1935-1939 | | | Cohort 1930-1934 | | | Cohort 1925-1929 | | | Cohort 1920-1924 | | |
| --- | --- | --- | --- | --- | --- | --- | --- | --- | --- | --- | --- | --- | --- | --- | --- | --- |
| Age | Sex | PP | LCI | UCI | PP | LCI | UCI | PP | LCI | UCI | PP | LCI | UCI | PP | LCI | UCI |
| 62 | M | 0.062 | 0.044 | 0.081 |  |  |  |  |  |  |  |  |  |  |  |  |
| 62 | W | 0.062 | 0.044 | 0.080 |  |  |  |  |  |  |  |  |  |  |  |  |
| … |  |  |  |  |  |  |  |  |  |  |  |  |  |  |  |  |
| 65 | M | 0.067 | 0.051 | 0.084 |  |  |  |  |  |  |  |  |  |  |  |  |
| 65 | W | 0.063 | 0.048 | 0.079 |  |  |  |  |  |  |  |  |  |  |  |  |
| 66 | M |  |  |  |  |  |  |  |  |  |  |  |  |  |  |  |
| 66 | W |  |  |  |  |  |  |  |  |  |  |  |  |  |  |  |
| 67 | M |  |  |  | 0.059 | 0.046 | 0.073 |  |  |  |  |  |  |  |  |  |
| 67 | W |  |  |  | 0.062 | 0.048 | 0.076 |  |  |  |  |  |  |  |  |  |
| 68 | M |  |  |  |  |  |  |  |  |  |  |  |  |  |  |  |
| 68 | W |  |  |  |  |  |  |  |  |  |  |  |  |  |  |  |
| 69 | M | 0.084 | 0.066 | 0.102 |  |  |  |  |  |  |  |  |  |  |  |  |
| 69 | W | 0.071 | 0.055 | 0.087 |  |  |  |  |  |  |  |  |  |  |  |  |
| 70 | M |  |  |  | 0.072 | 0.059 | 0.084 |  |  |  |  |  |  |  |  |  |
| 70 | W |  |  |  | 0.070 | 0.057 | 0.083 |  |  |  |  |  |  |  |  |  |
| 71 | M | 0.094 | 0.075 | 0.114 |  |  |  |  |  |  |  |  |  |  |  |  |
| 71 | W | 0.077 | 0.061 | 0.093 |  |  |  |  |  |  |  |  |  |  |  |  |
| 72 | M |  |  |  |  |  |  | 0.066 | 0.050 | 0.083 |  |  |  |  |  |  |
| 72 | W |  |  |  |  |  |  | 0.072 | 0.056 | 0.089 |  |  |  |  |  |  |
| 73 | M | 0.105 | 0.084 | 0.127 |  |  |  |  |  |  |  |  |  |  |  |  |
| 73 | W | 0.084 | 0.066 | 0.101 |  |  |  |  |  |  |  |  |  |  |  |  |
| 74 | M |  |  |  | 0.107 | 0.090 | 0.123 |  |  |  |  |  |  |  |  |  |
| 74 | W |  |  |  | 0.095 | 0.080 | 0.110 |  |  |  |  |  |  |  |  |  |
| 75 | M | 0.116 | 0.091 | 0.141 |  |  |  | 0.089 | 0.072 | 0.106 |  |  |  |  |  |  |
| 75 | W | 0.090 | 0.070 | 0.111 |  |  |  | 0.091 | 0.074 | 0.108 |  |  |  |  |  |  |
| 76 | M |  |  |  | 0.127 | 0.109 | 0.146 |  |  |  |  |  |  |  |  |  |
| 76 | W |  |  |  | 0.110 | 0.094 | 0.125 |  |  |  |  |  |  |  |  |  |
| 77 | M |  |  |  |  |  |  |  |  |  | 0.087 | 0.063 | 0.110 |  |  |  |
| 77 | W |  |  |  |  |  |  |  |  |  | 0.098 | 0.076 | 0.120 |  |  |  |
| 78 | M |  |  |  | 0.149 | 0.128 | 0.170 |  |  |  |  |  |  |  |  |  |
| 78 | W |  |  |  | 0.125 | 0.107 | 0.142 |  |  |  |  |  |  |  |  |  |
| 79 | M |  |  |  |  |  |  | 0.151 | 0.128 | 0.173 |  |  |  |  |  |  |
| 79 | W |  |  |  |  |  |  | 0.140 | 0.119 | 0.161 |  |  |  |  |  |  |
| 80 | M |  |  |  | 0.170 | 0.143 | 0.197 |  |  |  | 0.125 | 0.100 | 0.150 |  |  |  |
| 80 | W |  |  |  | 0.140 | 0.118 | 0.162 |  |  |  | 0.133 | 0.110 | 0.155 |  |  |  |
| 81 | M |  |  |  |  |  |  | 0.187 | 0.161 | 0.213 |  |  |  |  |  |  |
| 81 | W |  |  |  |  |  |  | 0.168 | 0.145 | 0.192 |  |  |  |  |  |  |
| 82 | M |  |  |  |  |  |  |  |  |  |  |  |  | 0.129 | 0.082 | 0.176 |
| 82 | W |  |  |  |  |  |  |  |  |  |  |  |  | 0.150 | 0.107 | 0.193 |
| 83 | M |  |  |  |  |  |  | 0.224 | 0.195 | 0.254 |  |  |  |  |  |  |
| 83 | W |  |  |  |  |  |  | 0.197 | 0.171 | 0.224 |  |  |  |  |  |  |
| 84 | M |  |  |  |  |  |  |  |  |  | 0.228 | 0.195 | 0.262 |  |  |  |
| 84 | W |  |  |  |  |  |  |  |  |  | 0.221 | 0.191 | 0.250 |  |  |  |
| 85 | M |  |  |  |  |  |  | 0.260 | 0.224 | 0.297 |  |  |  | 0.193 | 0.140 | 0.246 |
| 85 | W |  |  |  |  |  |  | 0.226 | 0.193 | 0.259 |  |  |  | 0.210 | 0.163 | 0.256 |
| 86 | M |  |  |  |  |  |  |  |  |  | 0.286 | 0.248 | 0.324 |  |  |  |
| 86 | W |  |  |  |  |  |  |  |  |  | 0.269 | 0.235 | 0.302 |  |  |  |
| 87 | M |  |  |  |  |  |  |  |  |  |  |  |  |  |  |  |
| 87 | W |  |  |  |  |  |  |  |  |  |  |  |  |  |  |  |
| 88 | M |  |  |  |  |  |  |  |  |  | 0.341 | 0.298 | 0.384 |  |  |  |
| 88 | W |  |  |  |  |  |  |  |  |  | 0.315 | 0.276 | 0.353 |  |  |  |
| 89 | M |  |  |  |  |  |  |  |  |  |  |  |  | 0.352 | 0.286 | 0.418 |
| 89 | W |  |  |  |  |  |  |  |  |  |  |  |  | 0.351 | 0.293 | 0.408 |
| 90 | M |  |  |  |  |  |  |  |  |  | 0.391 | 0.339 | 0.444 |  |  |  |
| 90 | W |  |  |  |  |  |  |  |  |  | 0.356 | 0.309 | 0.404 |  |  |  |
| 91 | M |  |  |  |  |  |  |  |  |  |  |  |  | 0.432 | 0.361 | 0.502 |
| 91 | W |  |  |  |  |  |  |  |  |  |  |  |  | 0.419 | 0.357 | 0.482 |
| 92 | M |  |  |  |  |  |  |  |  |  |  |  |  |  |  |  |
| 92 | W |  |  |  |  |  |  |  |  |  |  |  |  |  |  |  |
| 93 | M |  |  |  |  |  |  |  |  |  |  |  |  | 0.503 | 0.428 | 0.578 |
| 93 | W |  |  |  |  |  |  |  |  |  |  |  |  | 0.481 | 0.412 | 0.549 |
| 94 | M |  |  |  |  |  |  |  |  |  |  |  |  |  |  |  |
| 94 | W |  |  |  |  |  |  |  |  |  |  |  |  | 0.534 | 0.458 | 0.610 |
| 95 | M |  |  |  |  |  |  |  |  |  |  |  |  | 0.564 | 0.484 | 0.644 |

**Supplementary table 9.** Predicted probabilities of **ADL** limitations by age and sex adjusted for income and education (**model 2**)**. Southern Europe**, data for figure 1. (PP, predicted probability. LCI, lower confidence interval. UCI, upper confidence interval).

|  |  | Cohort 1940-1944 | | | Cohort 1935-1939 | | | Cohort 1930-1934 | | | Cohort 1925-1929 | | | Cohort 1920-1924 | | |
| --- | --- | --- | --- | --- | --- | --- | --- | --- | --- | --- | --- | --- | --- | --- | --- | --- |
| Age | Sex | PP | LCI | UCI | PP | LCI | UCI | PP | LCI | UCI | PP | LCI | UCI | PP | LCI | UCI |
| 62 | M | 0.046 | 0.032 | 0.060 |  |  |  |  |  |  |  |  |  |  |  |  |
| 62 | W | 0.069 | 0.052 | 0.085 |  |  |  |  |  |  |  |  |  |  |  |  |
| … |  |  |  |  |  |  |  |  |  |  |  |  |  |  |  |  |
| 65 | M | 0.048 | 0.036 | 0.061 |  |  |  |  |  |  |  |  |  |  |  |  |
| 65 | W | 0.073 | 0.059 | 0.088 |  |  |  |  |  |  |  |  |  |  |  |  |
| 66 | M |  |  |  |  |  |  |  |  |  |  |  |  |  |  |  |
| 66 | W |  |  |  |  |  |  |  |  |  |  |  |  |  |  |  |
| 67 | M |  |  |  | 0.056 | 0.044 | 0.067 |  |  |  |  |  |  |  |  |  |
| 67 | W |  |  |  | 0.083 | 0.069 | 0.097 |  |  |  |  |  |  |  |  |  |
| 68 | M |  |  |  |  |  |  |  |  |  |  |  |  |  |  |  |
| 68 | W |  |  |  |  |  |  |  |  |  |  |  |  |  |  |  |
| 69 | M | 0.061 | 0.047 | 0.075 |  |  |  |  |  |  |  |  |  |  |  |  |
| 69 | W | 0.093 | 0.077 | 0.109 |  |  |  |  |  |  |  |  |  |  |  |  |
| 70 | M |  |  |  | 0.065 | 0.055 | 0.076 |  |  |  |  |  |  |  |  |  |
| 70 | W |  |  |  | 0.098 | 0.085 | 0.111 |  |  |  |  |  |  |  |  |  |
| 71 | M | 0.071 | 0.055 | 0.087 |  |  |  |  |  |  |  |  |  |  |  |  |
| 71 | W | 0.107 | 0.090 | 0.125 |  |  |  |  |  |  |  |  |  |  |  |  |
| 72 | M |  |  |  |  |  |  | 0.079 | 0.064 | 0.095 |  |  |  |  |  |  |
| 72 | W |  |  |  |  |  |  | 0.116 | 0.098 | 0.134 |  |  |  |  |  |  |
| 73 | M | 0.084 | 0.066 | 0.102 |  |  |  |  |  |  |  |  |  |  |  |  |
| 73 | W | 0.124 | 0.104 | 0.144 |  |  |  |  |  |  |  |  |  |  |  |  |
| 74 | M |  |  |  | 0.097 | 0.084 | 0.111 |  |  |  |  |  |  |  |  |  |
| 74 | W |  |  |  | 0.143 | 0.127 | 0.159 |  |  |  |  |  |  |  |  |  |
| 75 | M | 0.098 | 0.077 | 0.120 |  |  |  | 0.102 | 0.086 | 0.117 |  |  |  |  |  |  |
| 75 | W | 0.143 | 0.118 | 0.167 |  |  |  | 0.148 | 0.129 | 0.166 |  |  |  |  |  |  |
| 76 | M |  |  |  | 0.120 | 0.104 | 0.135 |  |  |  |  |  |  |  |  |  |
| 76 | W |  |  |  | 0.172 | 0.154 | 0.190 |  |  |  |  |  |  |  |  |  |
| 77 | M |  |  |  |  |  |  |  |  |  | 0.128 | 0.101 | 0.155 |  |  |  |
| 77 | W |  |  |  |  |  |  |  |  |  | 0.181 | 0.154 | 0.208 |  |  |  |
| 78 | M |  |  |  | 0.145 | 0.127 | 0.164 |  |  |  |  |  |  |  |  |  |
| 78 | W |  |  |  | 0.205 | 0.185 | 0.225 |  |  |  |  |  |  |  |  |  |
| 79 | M |  |  |  |  |  |  | 0.168 | 0.147 | 0.188 |  |  |  |  |  |  |
| 79 | W |  |  |  |  |  |  | 0.233 | 0.209 | 0.258 |  |  |  |  |  |  |
| 80 | M |  |  |  | 0.174 | 0.150 | 0.199 |  |  |  | 0.173 | 0.145 | 0.201 |  |  |  |
| 80 | W |  |  |  | 0.240 | 0.214 | 0.267 |  |  |  | 0.238 | 0.210 | 0.266 |  |  |  |
| 81 | M |  |  |  |  |  |  | 0.210 | 0.187 | 0.233 |  |  |  |  |  |  |
| 81 | W |  |  |  |  |  |  | 0.285 | 0.259 | 0.311 |  |  |  |  |  |  |
| 82 | M |  |  |  |  |  |  |  |  |  |  |  |  | 0.221 | 0.162 | 0.280 |
| 82 | W |  |  |  |  |  |  |  |  |  |  |  |  | 0.294 | 0.237 | 0.351 |
| 83 | M |  |  |  |  |  |  | 0.257 | 0.230 | 0.284 |  |  |  |  |  |  |
| 83 | W |  |  |  |  |  |  | 0.338 | 0.310 | 0.366 |  |  |  |  |  |  |
| 84 | M |  |  |  |  |  |  |  |  |  | 0.293 | 0.258 | 0.328 |  |  |  |
| 84 | W |  |  |  |  |  |  |  |  |  | 0.380 | 0.346 | 0.415 |  |  |  |
| 85 | M |  |  |  |  |  |  | 0.305 | 0.270 | 0.340 |  |  |  | 0.300 | 0.236 | 0.364 |
| 85 | W |  |  |  |  |  |  | 0.391 | 0.356 | 0.425 |  |  |  | 0.387 | 0.327 | 0.448 |
| 86 | M |  |  |  |  |  |  |  |  |  | 0.361 | 0.323 | 0.399 |  |  |  |
| 86 | W |  |  |  |  |  |  |  |  |  | 0.455 | 0.418 | 0.491 |  |  |  |
| 87 | M |  |  |  |  |  |  |  |  |  |  |  |  |  |  |  |
| 87 | W |  |  |  |  |  |  |  |  |  |  |  |  |  |  |  |
| 88 | M |  |  |  |  |  |  |  |  |  | 0.429 | 0.387 | 0.472 |  |  |  |
| 88 | W |  |  |  |  |  |  |  |  |  | 0.526 | 0.487 | 0.564 |  |  |  |
| 89 | M |  |  |  |  |  |  |  |  |  |  |  |  | 0.483 | 0.414 | 0.551 |
| 89 | W |  |  |  |  |  |  |  |  |  |  |  |  | 0.580 | 0.520 | 0.641 |
| 90 | M |  |  |  |  |  |  |  |  |  | 0.495 | 0.444 | 0.546 |  |  |  |
| 90 | W |  |  |  |  |  |  |  |  |  | 0.589 | 0.545 | 0.634 | 0.663 | 0.606 | 0.720 |
| 91 | M |  |  |  |  |  |  |  |  |  |  |  |  | 0.570 | 0.502 | 0.637 |
| 91 | W |  |  |  |  |  |  |  |  |  |  |  |  |  |  |  |
| 92 | M |  |  |  |  |  |  |  |  |  |  |  |  | 0.646 | 0.580 | 0.711 |
| 92 | W |  |  |  |  |  |  |  |  |  |  |  |  |  |  |  |
| 93 | M |  |  |  |  |  |  |  |  |  |  |  |  |  |  |  |
| 93 | W |  |  |  |  |  |  |  |  |  |  |  |  | 0.731 | 0.677 | 0.785 |
| 94 | M |  |  |  |  |  |  |  |  |  |  |  |  | 0.710 | 0.644 | 0.775 |
| 94 | W |  |  |  |  |  |  |  |  |  |  |  |  | 0.786 | 0.734 | 0.839 |

**Supplementary table 10.** Predicted probabilities of **ADL** limitations by age and sex adjusted for income and education (**model 2**). **Western Europe**, data for figure 1. (PP, predicted probability. LCI, lower confidence interval. UCI, upper confidence interval).

|  |  | Cohort 1940-1944 | | | Cohort 1935-1939 | | | Cohort 1930-1934 | | | Cohort 1925-1929 | | | Cohort 1920-1924 | | |
| --- | --- | --- | --- | --- | --- | --- | --- | --- | --- | --- | --- | --- | --- | --- | --- | --- |
| Age | Sex | PP | LCI | UCI | PP | LCI | UCI | PP | LCI | UCI | PP | LCI | UCI | PP | LCI | UCI |
| 62 | M | 0.064 | 0.047 | 0.081 |  |  |  |  |  |  |  |  |  |  |  |  |
| 62 | W | 0.070 | 0.054 | 0.086 |  |  |  |  |  |  |  |  |  |  |  |  |
| … |  |  |  |  |  |  |  |  |  |  |  |  |  |  |  |  |
| 65 | M | 0.074 | 0.057 | 0.090 |  |  |  |  |  |  |  |  |  |  |  |  |
| 65 | W | 0.077 | 0.062 | 0.092 |  |  |  |  |  |  |  |  |  |  |  |  |
| 66 | M |  |  |  |  |  |  |  |  |  |  |  |  |  |  |  |
| 66 | W |  |  |  |  |  |  |  |  |  |  |  |  |  |  |  |
| 67 | M |  |  |  | 0.067 | 0.054 | 0.079 |  |  |  |  |  |  |  |  |  |
| 67 | W |  |  |  | 0.074 | 0.062 | 0.086 |  |  |  |  |  |  |  |  |  |
| 68 | M |  |  |  |  |  |  |  |  |  |  |  |  |  |  |  |
| 68 | W |  |  |  |  |  |  |  |  |  |  |  |  |  |  |  |
| 69 | M | 0.103 | 0.084 | 0.121 |  |  |  |  |  |  |  |  |  |  |  |  |
| 69 | W | 0.099 | 0.083 | 0.116 |  |  |  |  |  |  |  |  |  |  |  |  |
| 70 | M |  |  |  | 0.084 | 0.072 | 0.096 |  |  |  |  |  |  |  |  |  |
| 70 | W |  |  |  | 0.089 | 0.078 | 0.101 |  |  |  |  |  |  |  |  |  |
| 71 | M | 0.120 | 0.100 | 0.141 |  |  |  |  |  |  |  |  |  |  |  |  |
| 71 | W | 0.113 | 0.096 | 0.131 |  |  |  |  |  |  |  |  |  |  |  |  |
| 72 | M |  |  |  |  |  |  | 0.082 | 0.068 | 0.097 |  |  |  |  |  |  |
| 72 | W |  |  |  |  |  |  | 0.093 | 0.078 | 0.107 |  |  |  |  |  |  |
| 73 | M | 0.140 | 0.116 | 0.163 |  |  |  |  |  |  |  |  |  |  |  |  |
| 73 | W | 0.129 | 0.108 | 0.149 |  |  |  |  |  |  |  |  |  |  |  |  |
| 74 | M |  |  |  | 0.134 | 0.118 | 0.149 |  |  |  |  |  |  |  |  |  |
| 74 | W |  |  |  | 0.132 | 0.117 | 0.146 |  |  |  |  |  |  |  |  |  |
| 75 | M | 0.160 | 0.131 | 0.189 |  |  |  | 0.112 | 0.097 | 0.127 |  |  |  |  |  |  |
| 75 | W | 0.144 | 0.119 | 0.170 |  |  |  | 0.120 | 0.105 | 0.136 |  |  |  |  |  |  |
| 76 | M |  |  |  | 0.164 | 0.146 | 0.182 |  |  |  |  |  |  |  |  |  |
| 76 | W |  |  |  | 0.157 | 0.141 | 0.173 |  |  |  |  |  |  |  |  |  |
| 77 | M |  |  |  |  |  |  |  |  |  | 0.119 | 0.098 | 0.140 |  |  |  |
| 77 | W |  |  |  |  |  |  |  |  |  | 0.134 | 0.114 | 0.154 |  |  |  |
| 78 | M |  |  |  | 0.197 | 0.174 | 0.219 |  |  |  |  |  |  |  |  |  |
| 78 | W |  |  |  | 0.184 | 0.165 | 0.204 |  |  |  |  |  |  |  |  |  |
| 79 | M |  |  |  |  |  |  | 0.193 | 0.173 | 0.213 |  |  |  |  |  |  |
| 79 | W |  |  |  |  |  |  | 0.193 | 0.172 | 0.213 |  |  |  |  |  |  |
| 80 | M |  |  |  | 0.231 | 0.201 | 0.260 |  |  |  | 0.168 | 0.146 | 0.191 |  |  |  |
| 80 | W |  |  |  | 0.213 | 0.186 | 0.239 |  |  |  | 0.181 | 0.160 | 0.202 |  |  |  |
| 81 | M |  |  |  |  |  |  | 0.242 | 0.218 | 0.266 |  |  |  |  |  |  |
| 81 | W |  |  |  |  |  |  | 0.235 | 0.212 | 0.258 |  |  |  |  |  |  |
| 82 | M |  |  |  |  |  |  |  |  |  |  |  |  | 0.190 | 0.146 | 0.234 |
| 82 | W |  |  |  |  |  |  |  |  |  |  |  |  | 0.213 | 0.174 | 0.251 |
| 83 | M |  |  |  |  |  |  | 0.292 | 0.263 | 0.321 |  |  |  |  |  |  |
| 83 | W |  |  |  |  |  |  | 0.279 | 0.252 | 0.306 |  |  |  |  |  |  |
| 84 | M |  |  |  |  |  |  |  |  |  | 0.296 | 0.264 | 0.327 |  |  |  |
| 84 | W |  |  |  |  |  |  |  |  |  | 0.298 | 0.269 | 0.326 |  |  |  |
| 85 | M |  |  |  |  |  |  | 0.340 | 0.304 | 0.377 |  |  |  | 0.269 | 0.220 | 0.318 |
| 85 | W |  |  |  |  |  |  | 0.321 | 0.288 | 0.355 |  |  |  | 0.288 | 0.246 | 0.329 |
| 86 | M |  |  |  |  |  |  |  |  |  | 0.366 | 0.329 | 0.402 |  |  |  |
| 86 | W |  |  |  |  |  |  |  |  |  | 0.361 | 0.328 | 0.393 |  |  |  |
| 87 | M |  |  |  |  |  |  |  |  |  |  |  |  |  |  |  |
| 87 | W |  |  |  |  |  |  |  |  |  |  |  |  |  |  |  |
| 88 | M |  |  |  |  |  |  |  |  |  | 0.433 | 0.390 | 0.476 |  |  |  |
| 88 | W |  |  |  |  |  |  |  |  |  | 0.421 | 0.383 | 0.459 |  |  |  |
| 89 | M |  |  |  |  |  |  |  |  |  | 0.495 | 0.444 | 0.547 | 0.452 | 0.392 | 0.512 |
| 89 | W |  |  |  |  |  |  |  |  |  |  |  |  | 0.457 | 0.406 | 0.509 |
| 90 | M |  |  |  |  |  |  |  |  |  |  |  |  | 0.539 | 0.476 | 0.602 |
| 90 | W |  |  |  |  |  |  |  |  |  | 0.477 | 0.430 | 0.523 | 0.537 | 0.482 | 0.591 |
| 91 | M |  |  |  |  |  |  |  |  |  |  |  |  |  |  |  |
| 91 | W |  |  |  |  |  |  |  |  |  |  |  |  |  |  |  |
| 92 | M |  |  |  |  |  |  |  |  |  |  |  |  | 0.615 | 0.552 | 0.679 |
| 92 | W |  |  |  |  |  |  |  |  |  |  |  |  | 0.606 | 0.549 | 0.662 |
| 93 | M |  |  |  |  |  |  |  |  |  |  |  |  |  |  |  |
| 93 | W |  |  |  |  |  |  |  |  |  |  |  |  |  |  |  |
| 94 | M |  |  |  |  |  |  |  |  |  |  |  |  | 0.677 | 0.613 | 0.742 |
| 94 | W |  |  |  |  |  |  |  |  |  |  |  |  | 0.663 | 0.604 | 0.722 |

**Supplementary table 11.** Predicted probabilities of **IADL** limitations by age and sex (**model 1**). **Eastern Europe**, data for figure 2. (PP, predicted probability. LCI, lower confidence interval. UCI, upper confidence interval).

|  |  | Cohort 1940-1944 | | | Cohort 1935-1939 | | | Cohort 1930-1934 | | | Cohort 1925-1929 | | | Cohort 1920-1924 | | |
| --- | --- | --- | --- | --- | --- | --- | --- | --- | --- | --- | --- | --- | --- | --- | --- | --- |
| Age | Sex | PP | LCI | UCI | PP | LCI | UCI | PP | LCI | UCI | PP | LCI | UCI | PP | LCI | UCI |
| … |  |  |  |  |  |  |  |  |  |  |  |  |  |  |  |  |
| 65 | M | 0.140 | 0.097 | 0.184 |  |  |  |  |  |  |  |  |  |  |  |  |
| 65 | W | 0.284 | 0.221 | 0.347 |  |  |  |  |  |  |  |  |  |  |  |  |
| 66 | M |  |  |  |  |  |  |  |  |  |  |  |  |  |  |  |
| 66 | W |  |  |  |  |  |  |  |  |  |  |  |  |  |  |  |
| 67 | M |  |  |  |  |  |  |  |  |  |  |  |  |  |  |  |
| 67 | W |  |  |  |  |  |  |  |  |  |  |  |  |  |  |  |
| 68 | M |  |  |  |  |  |  |  |  |  |  |  |  |  |  |  |
| 68 | W |  |  |  |  |  |  |  |  |  |  |  |  |  |  |  |
| 69 | M | 0.137 | 0.100 | 0.174 |  |  |  |  |  |  |  |  |  |  |  |  |
| 69 | W | 0.251 | 0.203 | 0.299 |  |  |  |  |  |  |  |  |  |  |  |  |
| 70 | M |  |  |  | 0.181 | 0.142 | 0.219 |  |  |  |  |  |  |  |  |  |
| 70 | W |  |  |  | 0.351 | 0.302 | 0.400 |  |  |  |  |  |  |  |  |  |
| 71 | M | 0.151 | 0.111 | 0.191 |  |  |  |  |  |  |  |  |  |  |  |  |
| 71 | W | 0.257 | 0.210 | 0.303 |  |  |  |  |  |  |  |  |  |  |  |  |
| 72 | M |  |  |  |  |  |  |  |  |  |  |  |  |  |  |  |
| 72 | W |  |  |  |  |  |  |  |  |  |  |  |  |  |  |  |
| 73 | M | 0.174 | 0.129 | 0.220 |  |  |  |  |  |  |  |  |  |  |  |  |
| 73 | W | 0.275 | 0.227 | 0.323 |  |  |  |  |  |  |  |  |  |  |  |  |
| 74 | M |  |  |  | 0.208 | 0.172 | 0.244 |  |  |  |  |  |  |  |  |  |
| 74 | W |  |  |  | 0.356 | 0.314 | 0.398 |  |  |  |  |  |  |  |  |  |
| 75 | M | 0.208 | 0.152 | 0.264 |  |  |  | 0.236 | 0.185 | 0.287 |  |  |  |  |  |  |
| 75 | W | 0.304 | 0.247 | 0.361 |  |  |  | 0.434 | 0.377 | 0.492 |  |  |  |  |  |  |
| 76 | M |  |  |  | 0.241 | 0.200 | 0.281 |  |  |  |  |  |  |  |  |  |
| 76 | W |  |  |  | 0.380 | 0.339 | 0.422 |  |  |  |  |  |  |  |  |  |
| 77 | M |  |  |  |  |  |  |  |  |  |  |  |  |  |  |  |
| 77 | W |  |  |  |  |  |  |  |  |  |  |  |  |  |  |  |
| 78 | M |  |  |  | 0.285 | 0.239 | 0.332 |  |  |  |  |  |  |  |  |  |
| 78 | W |  |  |  | 0.417 | 0.375 | 0.458 |  |  |  |  |  |  |  |  |  |
| 79 | M |  |  |  |  |  |  | 0.307 | 0.256 | 0.357 |  |  |  |  |  |  |
| 79 | W |  |  |  |  |  |  | 0.484 | 0.434 | 0.534 |  |  |  |  |  |  |
| 80 | M |  |  |  | 0.341 | 0.282 | 0.399 |  |  |  | 0.309 | 0.230 | 0.387 |  |  |  |
| 80 | W |  |  |  | 0.463 | 0.411 | 0.514 |  |  |  | 0.531 | 0.457 | 0.604 |  |  |  |
| 81 | M |  |  |  |  |  |  | 0.363 | 0.308 | 0.418 |  |  |  |  |  |  |
| 81 | W |  |  |  |  |  |  | 0.527 | 0.478 | 0.576 |  |  |  |  |  |  |
| 82 | M |  |  |  |  |  |  |  |  |  |  |  |  |  |  |  |
| 82 | W |  |  |  |  |  |  |  |  |  |  |  |  |  |  |  |
| 83 | M |  |  |  |  |  |  | 0.431 | 0.371 | 0.491 |  |  |  |  |  |  |
| 83 | W |  |  |  |  |  |  | 0.579 | 0.530 | 0.628 |  |  |  |  |  |  |
| 84 | M |  |  |  |  |  |  |  |  |  | 0.432 | 0.357 | 0.507 | 0.400 | 0.257 | 0.543 |
| 84 | W |  |  |  |  |  |  |  |  |  | 0.622 | 0.564 | 0.681 |  |  |  |
| 85 | M |  |  |  |  |  |  | 0.506 | 0.437 | 0.576 |  |  |  |  |  |  |
| 85 | W |  |  |  |  |  |  | 0.636 | 0.580 | 0.691 |  |  |  | 0.635 | 0.514 | 0.755 |
| 86 | M |  |  |  |  |  |  |  |  |  | 0.512 | 0.435 | 0.588 |  |  |  |
| 86 | W |  |  |  |  |  |  |  |  |  | 0.678 | 0.622 | 0.734 |  |  |  |
| 87 | M |  |  |  |  |  |  |  |  |  |  |  |  |  |  |  |
| 87 | W |  |  |  |  |  |  |  |  |  |  |  |  |  |  |  |
| 88 | M |  |  |  |  |  |  |  |  |  | 0.595 | 0.518 | 0.673 | 0.574 | 0.446 | 0.702 |
| 88 | W |  |  |  |  |  |  |  |  |  | 0.735 | 0.681 | 0.790 |  |  |  |
| 89 | M |  |  |  |  |  |  |  |  |  |  |  |  |  |  |  |
| 89 | W |  |  |  |  |  |  |  |  |  |  |  |  | 0.753 | 0.666 | 0.841 |
| 90 | M |  |  |  |  |  |  |  |  |  | 0.677 | 0.597 | 0.758 | 0.666 | 0.550 | 0.782 |
| 90 | W |  |  |  |  |  |  |  |  |  | 0.789 | 0.733 | 0.846 | 0.809 | 0.735 | 0.884 |
| 91 | M |  |  |  |  |  |  |  |  |  |  |  |  |  |  |  |
| 91 | W |  |  |  |  |  |  |  |  |  |  |  |  |  |  |  |
| 92 | M |  |  |  |  |  |  |  |  |  |  |  |  | 0.750 | 0.648 | 0.853 |
| 92 | W |  |  |  |  |  |  |  |  |  |  |  |  | 0.858 | 0.795 | 0.922 |
| 93 | M |  |  |  |  |  |  |  |  |  |  |  |  |  |  |  |
| 93 | W |  |  |  |  |  |  |  |  |  |  |  |  |  |  |  |
| 94 | M |  |  |  |  |  |  |  |  |  |  |  |  | 0.822 | 0.733 | 0.910 |
| 94 | W |  |  |  |  |  |  |  |  |  |  |  |  |  |  |  |
| 95 | M |  |  |  |  |  |  |  |  |  |  |  |  |  |  |  |
| 95 | W |  |  |  |  |  |  |  |  |  |  |  |  | 0.899 | 0.844 | 0.953 |

**Supplementary table 12.** Predicted probabilities of **IADL** limitations by age and sex (**model 1**). **Northern Europe**, data for figure 2. (PP, predicted probability. LCI, lower confidence interval. UCI, upper confidence interval).

|  |  | Cohort 1940-1944 | | | Cohort 1935-1939 | | | Cohort 1930-1934 | | | Cohort 1925-1929 | | | Cohort 1920-1924 | | |
| --- | --- | --- | --- | --- | --- | --- | --- | --- | --- | --- | --- | --- | --- | --- | --- | --- |
| Age | Sex | PP | LCI | UCI | PP | LCI | UCI | PP | LCI | UCI | PP | LCI | UCI | PP | LCI | UCI |
| 62 | M | 0.049 | 0.035 | 0.064 |  |  |  |  |  |  |  |  |  |  |  |  |
| 62 | W | 0.098 | 0.076 | 0.121 |  |  |  |  |  |  |  |  |  |  |  |  |
| … |  |  |  |  |  |  |  |  |  |  |  |  |  |  |  |  |
| 65 | M | 0.052 | 0.039 | 0.065 |  |  |  |  |  |  |  |  |  |  |  |  |
| 65 | W | 0.095 | 0.076 | 0.115 |  |  |  |  |  |  |  |  |  |  |  |  |
| 66 | M |  |  |  |  |  |  |  |  |  |  |  |  |  |  |  |
| 66 | W |  |  |  |  |  |  |  |  |  |  |  |  |  |  |  |
| 67 | M |  |  |  | 0.066 | 0.051 | 0.080 |  |  |  |  |  |  |  |  |  |
| 67 | W |  |  |  | 0.125 | 0.105 | 0.145 |  |  |  |  |  |  |  |  |  |
| 68 | M |  |  |  |  |  |  |  |  |  |  |  |  |  |  |  |
| 68 | W |  |  |  |  |  |  |  |  |  |  |  |  |  |  |  |
| 69 | M | 0.073 | 0.057 | 0.089 |  |  |  |  |  |  |  |  |  |  |  |  |
| 69 | W | 0.110 | 0.090 | 0.131 |  |  |  |  |  |  |  |  |  |  |  |  |
| 70 | M |  |  |  | 0.076 | 0.063 | 0.090 |  |  |  |  |  |  |  |  |  |
| 70 | W |  |  |  | 0.132 | 0.115 | 0.149 |  |  |  |  |  |  |  |  |  |
| 71 | M | 0.092 | 0.074 | 0.111 |  |  |  |  |  |  |  |  |  |  |  |  |
| 71 | W | 0.127 | 0.105 | 0.148 |  |  |  |  |  |  |  |  |  |  |  |  |
| 72 | M |  |  |  |  |  |  | 0.099 | 0.078 | 0.120 |  |  |  |  |  |  |
| 72 | W |  |  |  |  |  |  | 0.175 | 0.149 | 0.201 |  |  |  |  |  |  |
| 73 | M | 0.117 | 0.095 | 0.140 |  |  |  |  |  |  |  |  |  |  |  |  |
| 73 | W | 0.148 | 0.123 | 0.173 |  |  |  |  |  |  |  |  |  |  |  |  |
| 74 | M |  |  |  | 0.120 | 0.103 | 0.137 |  |  |  |  |  |  |  |  |  |
| 74 | W |  |  |  | 0.171 | 0.151 | 0.190 |  |  |  |  |  |  |  |  |  |
| 75 | M | 0.149 | 0.119 | 0.178 |  |  |  | 0.123 | 0.103 | 0.143 |  |  |  |  |  |  |
| 75 | W | 0.176 | 0.145 | 0.206 |  |  |  | 0.197 | 0.173 | 0.220 |  |  |  |  |  |  |
| 76 | M |  |  |  | 0.155 | 0.134 | 0.175 |  |  |  |  |  |  |  |  |  |
| 76 | W |  |  |  | 0.201 | 0.180 | 0.223 |  |  |  |  |  |  |  |  |  |
| 77 | M |  |  |  |  |  |  |  |  |  | 0.162 | 0.130 | 0.194 |  |  |  |
| 77 | W |  |  |  |  |  |  |  |  |  | 0.259 | 0.225 | 0.292 |  |  |  |
| 78 | M |  |  |  | 0.198 | 0.172 | 0.223 |  |  |  |  |  |  |  |  |  |
| 78 | W |  |  |  | 0.240 | 0.215 | 0.264 |  |  |  |  |  |  |  |  |  |
| 79 | M |  |  |  |  |  |  | 0.204 | 0.179 | 0.230 |  |  |  |  |  |  |
| 79 | W |  |  |  |  |  |  | 0.271 | 0.244 | 0.297 |  |  |  |  |  |  |
| 80 | M |  |  |  | 0.249 | 0.216 | 0.282 |  |  |  | 0.208 | 0.176 | 0.239 |  |  |  |
| 80 | W |  |  |  | 0.283 | 0.252 | 0.313 |  |  |  | 0.302 | 0.272 | 0.332 |  |  |  |
| 81 | M |  |  |  |  |  |  | 0.262 | 0.232 | 0.291 |  |  |  |  |  |  |
| 81 | W |  |  |  |  |  |  | 0.320 | 0.292 | 0.349 |  |  |  |  |  |  |
| 82 | M |  |  |  |  |  |  |  |  |  |  |  |  | 0.268 | 0.209 | 0.328 |
| 82 | W |  |  |  |  |  |  |  |  |  |  |  |  | 0.387 | 0.326 | 0.448 |
| 83 | M |  |  |  |  |  |  | 0.326 | 0.293 | 0.360 |  |  |  |  |  |  |
| 83 | W |  |  |  |  |  |  | 0.374 | 0.343 | 0.405 |  |  |  |  |  |  |
| 84 | M |  |  |  |  |  |  |  |  |  | 0.341 | 0.304 | 0.378 |  |  |  |
| 84 | W |  |  |  |  |  |  |  |  |  | 0.420 | 0.386 | 0.454 |  |  |  |
| 85 | M |  |  |  |  |  |  | 0.394 | 0.353 | 0.435 |  |  |  | 0.345 | 0.284 | 0.406 |
| 85 | W |  |  |  |  |  |  | 0.431 | 0.393 | 0.469 |  |  |  | 0.458 | 0.400 | 0.516 |
| 86 | M |  |  |  |  |  |  |  |  |  | 0.421 | 0.380 | 0.461 |  |  |  |
| 86 | W |  |  |  |  |  |  |  |  |  | 0.486 | 0.450 | 0.523 |  |  |  |
| 87 | M |  |  |  |  |  |  |  |  |  |  |  |  |  |  |  |
| 87 | W |  |  |  |  |  |  |  |  |  |  |  |  |  |  |  |
| 88 | M |  |  |  |  |  |  |  |  |  | 0.503 | 0.457 | 0.548 |  |  |  |
| 88 | W |  |  |  |  |  |  |  |  |  | 0.554 | 0.514 | 0.593 |  |  |  |
| 89 | M |  |  |  |  |  |  |  |  |  |  |  |  | 0.530 | 0.468 | 0.592 |
| 89 | W |  |  |  |  |  |  |  |  |  |  |  |  | 0.609 | 0.554 | 0.663 |
| 90 | M |  |  |  |  |  |  |  |  |  | 0.582 | 0.532 | 0.633 |  |  |  |
| 90 | W |  |  |  |  |  |  |  |  |  | 0.617 | 0.573 | 0.660 |  |  |  |
| 91 | M |  |  |  |  |  |  |  |  |  |  |  |  | 0.620 | 0.560 | 0.680 |
| 91 | W |  |  |  |  |  |  |  |  |  |  |  |  | 0.678 | 0.626 | 0.730 |
| 92 | M |  |  |  |  |  |  |  |  |  |  |  |  |  |  |  |
| 92 | W |  |  |  |  |  |  |  |  |  |  |  |  |  |  |  |
| 93 | M |  |  |  |  |  |  |  |  |  |  |  |  | 0.699 | 0.641 | 0.756 |
| 93 | W |  |  |  |  |  |  |  |  |  |  |  |  | 0.738 | 0.688 | 0.789 |
| 94 | M |  |  |  |  |  |  |  |  |  |  |  |  |  |  |  |
| 94 | W |  |  |  |  |  |  |  |  |  |  |  |  | 0.791 | 0.741 | 0.842 |
| 95 | M |  |  |  |  |  |  |  |  |  |  |  |  | 0.766 | 0.708 | 0.824 |

**Supplementary table 13.** Predicted probabilities of **IADL** limitations by age and sex (**model 1**). **Southern Europe**, data for figure 2. (PP, predicted probability. LCI, lower confidence interval. UCI, upper confidence interval).

|  |  | Cohort 1940-1944 | | | Cohort 1935-1939 | | | Cohort 1930-1934 | | | Cohort 1925-1929 | | | Cohort 1920-1924 | | |
| --- | --- | --- | --- | --- | --- | --- | --- | --- | --- | --- | --- | --- | --- | --- | --- | --- |
| Age | Sex | PP | LCI | UCI | PP | LCI | UCI | PP | LCI | UCI | PP | LCI | UCI | PP | LCI | UCI |
| 62 | M | 0.075 | 0.056 | 0.094 |  |  |  |  |  |  |  |  |  |  |  |  |
| 62 | W | 0.158 | 0.134 | 0.183 |  |  |  |  |  |  |  |  |  |  |  |  |
| … |  |  |  |  |  |  |  |  |  |  |  |  |  |  |  |  |
| 65 | M | 0.075 | 0.059 | 0.091 |  |  |  |  |  |  |  |  |  |  |  |  |
| 65 | W | 0.158 | 0.138 | 0.179 |  |  |  |  |  |  |  |  |  |  |  |  |
| 66 | M |  |  |  |  |  |  |  |  |  |  |  |  |  |  |  |
| 66 | W |  |  |  |  |  |  |  |  |  |  |  |  |  |  |  |
| 67 | M |  |  |  | 0.102 | 0.086 | 0.119 |  |  |  |  |  |  |  |  |  |
| 67 | W |  |  |  | 0.208 | 0.187 | 0.230 |  |  |  |  |  |  |  |  |  |
| 68 | M |  |  |  |  |  |  |  |  |  |  |  |  |  |  |  |
| 68 | W |  |  |  |  |  |  |  |  |  |  |  |  |  |  |  |
| 69 | M | 0.090 | 0.074 | 0.107 |  |  |  |  |  |  |  |  |  |  |  |  |
| 69 | W | 0.183 | 0.161 | 0.205 |  |  |  |  |  |  |  |  |  |  |  |  |
| 70 | M |  |  |  | 0.112 | 0.097 | 0.126 |  |  |  |  |  |  |  |  |  |
| 70 | W |  |  |  | 0.224 | 0.205 | 0.243 |  |  |  |  |  |  |  |  |  |
| 71 | M | 0.106 | 0.088 | 0.125 |  |  |  |  |  |  |  |  |  |  |  |  |
| 71 | W | 0.207 | 0.183 | 0.231 |  |  |  |  |  |  |  |  |  |  |  |  |
| 72 | M |  |  |  |  |  |  | 0.145 | 0.125 | 0.165 |  |  |  |  |  |  |
| 72 | W |  |  |  |  |  |  | 0.279 | 0.251 | 0.307 |  |  |  |  |  |  |
| 73 | M | 0.129 | 0.108 | 0.150 |  |  |  |  |  |  |  |  |  |  |  |  |
| 73 | W | 0.238 | 0.212 | 0.265 |  |  |  |  |  |  |  |  |  |  |  |  |
| 74 | M |  |  |  | 0.153 | 0.136 | 0.170 |  |  |  |  |  |  |  |  |  |
| 74 | W |  |  |  | 0.285 | 0.263 | 0.307 |  |  |  |  |  |  |  |  |  |
| 75 | M | 0.158 | 0.132 | 0.184 |  |  |  | 0.169 | 0.151 | 0.188 |  |  |  |  |  |  |
| 75 | W | 0.277 | 0.244 | 0.310 |  |  |  | 0.316 | 0.291 | 0.342 |  |  |  |  |  |  |
| 76 | M |  |  |  | 0.187 | 0.168 | 0.205 |  |  |  |  |  |  |  |  |  |
| 76 | W |  |  |  | 0.329 | 0.306 | 0.352 |  |  |  |  |  |  |  |  |  |
| 77 | M |  |  |  |  |  |  |  |  |  | 0.208 | 0.179 | 0.237 |  |  |  |
| 77 | W |  |  |  |  |  |  |  |  |  | 0.374 | 0.337 | 0.412 |  |  |  |
| 78 | M |  |  |  | 0.229 | 0.208 | 0.250 |  |  |  |  |  |  |  |  |  |
| 78 | W |  |  |  | 0.380 | 0.355 | 0.404 |  |  |  |  |  |  |  |  |  |
| 79 | M |  |  |  |  |  |  | 0.252 | 0.228 | 0.275 |  |  |  |  |  |  |
| 79 | W |  |  |  |  |  |  | 0.422 | 0.393 | 0.451 |  |  |  |  |  |  |
| 80 | M |  |  |  | 0.280 | 0.252 | 0.307 |  |  |  | 0.256 | 0.227 | 0.284 |  |  |  |
| 80 | W |  |  |  | 0.436 | 0.405 | 0.466 |  |  |  | 0.437 | 0.403 | 0.471 |  |  |  |
| 81 | M |  |  |  |  |  |  | 0.309 | 0.283 | 0.335 |  |  |  |  |  |  |
| 81 | W |  |  |  |  |  |  | 0.486 | 0.457 | 0.514 |  |  |  |  |  |  |
| 82 | M |  |  |  |  |  |  |  |  |  |  |  |  | 0.299 | 0.243 | 0.354 |
| 82 | W |  |  |  |  |  |  |  |  |  |  |  |  | 0.494 | 0.431 | 0.557 |
| 83 | M |  |  |  |  |  |  | 0.375 | 0.346 | 0.404 |  |  |  |  |  |  |
| 83 | W |  |  |  |  |  |  | 0.552 | 0.523 | 0.581 |  |  |  |  |  |  |
| 84 | M |  |  |  |  |  |  |  |  |  | 0.391 | 0.356 | 0.425 |  |  |  |
| 84 | W |  |  |  |  |  |  |  |  |  | 0.584 | 0.550 | 0.617 |  |  |  |
| 85 | M |  |  |  |  |  |  | 0.446 | 0.410 | 0.482 |  |  |  | 0.375 | 0.319 | 0.432 |
| 85 | W |  |  |  |  |  |  | 0.618 | 0.584 | 0.651 |  |  |  | 0.579 | 0.523 | 0.635 |
| 86 | M |  |  |  |  |  |  |  |  |  | 0.471 | 0.434 | 0.509 |  |  |  |
| 86 | W |  |  |  |  |  |  |  |  |  | 0.657 | 0.624 | 0.690 |  |  |  |
| 87 | M |  |  |  |  |  |  |  |  |  |  |  |  |  |  |  |
| 87 | W |  |  |  |  |  |  |  |  |  |  |  |  |  |  |  |
| 88 | M |  |  |  |  |  |  |  |  |  | 0.554 | 0.514 | 0.595 |  |  |  |
| 88 | W |  |  |  |  |  |  |  |  |  | 0.725 | 0.692 | 0.757 |  |  |  |
| 89 | M |  |  |  |  |  |  |  |  |  |  |  |  | 0.560 | 0.502 | 0.617 |
| 89 | W |  |  |  |  |  |  |  |  |  |  |  |  | 0.742 | 0.696 | 0.788 |
| 90 | M |  |  |  |  |  |  |  |  |  | 0.634 | 0.589 | 0.679 |  |  |  |
| 90 | W |  |  |  |  |  |  |  |  |  | 0.784 | 0.750 | 0.818 | 0.809 | 0.769 | 0.849 |
| 91 | M |  |  |  |  |  |  |  |  |  |  |  |  | 0.651 | 0.595 | 0.707 |
| 91 | W |  |  |  |  |  |  |  |  |  |  |  |  |  |  |  |
| 92 | M |  |  |  |  |  |  |  |  |  |  |  |  | 0.733 | 0.680 | 0.785 |
| 92 | W |  |  |  |  |  |  |  |  |  |  |  |  |  |  |  |
| 93 | M |  |  |  |  |  |  |  |  |  |  |  |  |  |  |  |
| 93 | W |  |  |  |  |  |  |  |  |  |  |  |  | 0.862 | 0.828 | 0.896 |
| 94 | M |  |  |  |  |  |  |  |  |  |  |  |  | 0.801 | 0.752 | 0.851 |
| 94 | W |  |  |  |  |  |  |  |  |  |  |  |  | 0.903 | 0.873 | 0.933 |

**Supplementary table 14.** Predicted probabilities of **IADL** limitations by age and sex (**model 1**). **Western Europe**, data for figure 2. (PP, predicted probability. LCI, lower confidence interval. UCI, upper confidence interval).

|  |  | Cohort 1940-1944 | | | Cohort 1935-1939 | | | Cohort 1930-1934 | | | Cohort 1925-1929 | | | Cohort 1920-1924 | | |
| --- | --- | --- | --- | --- | --- | --- | --- | --- | --- | --- | --- | --- | --- | --- | --- | --- |
| Age | Sex | PP | LCI | UCI | PP | LCI | UCI | PP | LCI | UCI | PP | LCI | UCI | PP | LCI | UCI |
| 62 | M | 0.064 | 0.049 | 0.080 |  |  |  |  |  |  |  |  |  |  |  |  |
| 62 | W | 0.107 | 0.089 | 0.124 |  |  |  |  |  |  |  |  |  |  |  |  |
| … |  |  |  |  |  |  |  |  |  |  |  |  |  |  |  |  |
| 65 | M | 0.071 | 0.056 | 0.086 |  |  |  |  |  |  |  |  |  |  |  |  |
| 65 | W | 0.116 | 0.099 | 0.132 |  |  |  |  |  |  |  |  |  |  |  |  |
| 66 | M |  |  |  |  |  |  |  |  |  |  |  |  |  |  |  |
| 66 | W |  |  |  |  |  |  |  |  |  |  |  |  |  |  |  |
| 67 | M |  |  |  | 0.080 | 0.068 | 0.093 |  |  |  |  |  |  |  |  |  |
| 67 | W |  |  |  | 0.133 | 0.118 | 0.148 |  |  |  |  |  |  |  |  |  |
| 68 | M |  |  |  |  |  |  |  |  |  |  |  |  |  |  |  |
| 68 | W |  |  |  |  |  |  |  |  |  |  |  |  |  |  |  |
| 69 | M | 0.093 | 0.077 | 0.110 |  |  |  |  |  |  |  |  |  |  |  |  |
| 69 | W | 0.145 | 0.125 | 0.164 |  |  |  |  |  |  |  |  |  |  |  |  |
| 70 | M |  |  |  | 0.097 | 0.085 | 0.110 |  |  |  |  |  |  |  |  |  |
| 70 | W |  |  |  | 0.156 | 0.141 | 0.171 |  |  |  |  |  |  |  |  |  |
| 71 | M | 0.109 | 0.091 | 0.128 |  |  |  |  |  |  |  |  |  |  |  |  |
| 71 | W | 0.164 | 0.142 | 0.185 |  |  |  |  |  |  |  |  |  |  |  |  |
| 72 | M |  |  |  |  |  |  | 0.112 | 0.096 | 0.128 |  |  |  |  |  |  |
| 72 | W |  |  |  |  |  |  | 0.181 | 0.161 | 0.201 |  |  |  |  |  |  |
| 73 | M | 0.128 | 0.107 | 0.149 |  |  |  |  |  |  |  |  |  |  |  |  |
| 73 | W | 0.185 | 0.161 | 0.210 |  |  |  |  |  |  |  |  |  |  |  |  |
| 74 | M |  |  |  | 0.147 | 0.131 | 0.163 |  |  |  |  |  |  |  |  |  |
| 74 | W |  |  |  | 0.218 | 0.200 | 0.237 |  |  |  |  |  |  |  |  |  |
| 75 | M | 0.149 | 0.123 | 0.175 |  |  |  | 0.146 | 0.130 | 0.163 |  |  |  |  |  |  |
| 75 | W | 0.209 | 0.179 | 0.239 |  |  |  | 0.225 | 0.204 | 0.245 |  |  |  |  |  |  |
| 76 | M |  |  |  | 0.179 | 0.161 | 0.197 |  |  |  |  |  |  |  |  |  |
| 76 | W |  |  |  | 0.256 | 0.236 | 0.276 |  |  |  |  |  |  |  |  |  |
| 77 | M |  |  |  |  |  |  |  |  |  | 0.170 | 0.146 | 0.194 |  |  |  |
| 77 | W |  |  |  |  |  |  |  |  |  | 0.259 | 0.234 | 0.285 |  |  |  |
| 78 | M |  |  |  | 0.215 | 0.194 | 0.237 |  |  |  |  |  |  |  |  |  |
| 78 | W |  |  |  | 0.296 | 0.273 | 0.319 |  |  |  |  |  |  |  |  |  |
| 79 | M |  |  |  |  |  |  | 0.238 | 0.216 | 0.260 |  |  |  |  |  |  |
| 79 | W |  |  |  |  |  |  | 0.333 | 0.308 | 0.358 |  |  |  |  |  |  |
| 80 | M |  |  |  | 0.255 | 0.226 | 0.284 |  |  |  | 0.229 | 0.204 | 0.254 |  |  |  |
| 80 | W |  |  |  | 0.337 | 0.308 | 0.366 |  |  |  | 0.331 | 0.306 | 0.356 |  |  |  |
| 81 | M |  |  |  |  |  |  | 0.293 | 0.268 | 0.319 |  |  |  |  |  |  |
| 81 | W |  |  |  |  |  |  | 0.393 | 0.367 | 0.419 |  |  |  |  |  |  |
| 82 | M |  |  |  |  |  |  |  |  |  |  |  |  | 0.264 | 0.217 | 0.311 |
| 82 | W |  |  |  |  |  |  |  |  |  |  |  |  | 0.378 | 0.332 | 0.424 |
| 83 | M |  |  |  |  |  |  | 0.352 | 0.322 | 0.381 |  |  |  |  |  |  |
| 83 | W |  |  |  |  |  |  | 0.452 | 0.423 | 0.481 |  |  |  |  |  |  |
| 84 | M |  |  |  |  |  |  |  |  |  | 0.378 | 0.344 | 0.411 |  |  |  |
| 84 | W |  |  |  |  |  |  |  |  |  | 0.493 | 0.464 | 0.522 |  |  |  |
| 85 | M |  |  |  |  |  |  | 0.410 | 0.373 | 0.447 |  |  |  | 0.357 | 0.306 | 0.408 |
| 85 | W |  |  |  |  |  |  | 0.509 | 0.474 | 0.545 |  |  |  | 0.483 | 0.438 | 0.527 |
| 86 | M |  |  |  |  |  |  |  |  |  | 0.458 | 0.421 | 0.495 |  |  |  |
| 86 | W |  |  |  |  |  |  |  |  |  | 0.570 | 0.539 | 0.601 |  |  |  |
| 87 | M |  |  |  |  |  |  |  |  |  |  |  |  |  |  |  |
| 87 | W |  |  |  |  |  |  |  |  |  |  |  |  |  |  |  |
| 88 | M |  |  |  |  |  |  |  |  |  | 0.535 | 0.494 | 0.576 |  |  |  |
| 88 | W |  |  |  |  |  |  |  |  |  | 0.640 | 0.606 | 0.673 |  |  |  |
| 89 | M |  |  |  |  |  |  |  |  |  | 0.605 | 0.559 | 0.652 | 0.562 | 0.507 | 0.617 |
| 89 | W |  |  |  |  |  |  |  |  |  |  |  |  | 0.677 | 0.635 | 0.718 |
| 90 | M |  |  |  |  |  |  |  |  |  |  |  |  | 0.653 | 0.599 | 0.707 |
| 90 | W |  |  |  |  |  |  |  |  |  | 0.699 | 0.661 | 0.737 | 0.753 | 0.714 | 0.792 |
| 91 | M |  |  |  |  |  |  |  |  |  |  |  |  |  |  |  |
| 91 | W |  |  |  |  |  |  |  |  |  |  |  |  |  |  |  |
| 92 | M |  |  |  |  |  |  |  |  |  |  |  |  | 0.729 | 0.677 | 0.782 |
| 92 | W |  |  |  |  |  |  |  |  |  |  |  |  | 0.813 | 0.776 | 0.851 |
| 93 | M |  |  |  |  |  |  |  |  |  |  |  |  |  |  |  |
| 93 | W |  |  |  |  |  |  |  |  |  |  |  |  |  |  |  |
| 94 | M |  |  |  |  |  |  |  |  |  |  |  |  | 0.791 | 0.740 | 0.843 |
| 94 | W |  |  |  |  |  |  |  |  |  |  |  |  | 0.859 | 0.823 | 0.895 |

**Supplementary table 15.** Predicted probabilities of **IADL** limitations by age and sex adjusted for income and education (**model 2**). **Eastern Europe**, data for figure 2. (PP, predicted probability. LCI, lower confidence interval. UCI, upper confidence interval).

|  |  | Cohort 1940-1944 | | | Cohort 1935-1939 | | | Cohort 1930-1934 | | | Cohort 1925-1929 | | | Cohort 1920-1924 | | |
| --- | --- | --- | --- | --- | --- | --- | --- | --- | --- | --- | --- | --- | --- | --- | --- | --- |
| Age | Sex | PP | LCI | UCI | PP | LCI | UCI | PP | LCI | UCI | PP | LCI | UCI | PP | LCI | UCI |
| … |  |  |  |  |  |  |  |  |  |  |  |  |  |  |  |  |
| 65 | M | 0.161 | 0.113 | 0.210 |  |  |  |  |  |  |  |  |  |  |  |  |
| 65 | W | 0.287 | 0.224 | 0.350 |  |  |  |  |  |  |  |  |  |  |  |  |
| 66 | M |  |  |  |  |  |  |  |  |  |  |  |  |  |  |  |
| 66 | W |  |  |  |  |  |  |  |  |  |  |  |  |  |  |  |
| 67 | M |  |  |  |  |  |  |  |  |  |  |  |  |  |  |  |
| 67 | W |  |  |  |  |  |  |  |  |  |  |  |  |  |  |  |
| 68 | M |  |  |  |  |  |  |  |  |  |  |  |  |  |  |  |
| 68 | W |  |  |  |  |  |  |  |  |  |  |  |  |  |  |  |
| 69 | M | 0.158 | 0.117 | 0.198 |  |  |  |  |  |  |  |  |  |  |  |  |
| 69 | W | 0.255 | 0.207 | 0.303 |  |  |  |  |  |  |  |  |  |  |  |  |
| 70 | M |  |  |  | 0.197 | 0.156 | 0.238 |  |  |  |  |  |  |  |  |  |
| 70 | W |  |  |  | 0.339 | 0.291 | 0.386 |  |  |  |  |  |  |  |  |  |
| 71 | M | 0.171 | 0.128 | 0.214 |  |  |  |  |  |  |  |  |  |  |  |  |
| 71 | W | 0.260 | 0.213 | 0.306 |  |  |  |  |  |  |  |  |  |  |  |  |
| 72 | M |  |  |  |  |  |  |  |  |  |  |  |  |  |  |  |
| 72 | W |  |  |  |  |  |  |  |  |  |  |  |  |  |  |  |
| 73 | M | 0.194 | 0.146 | 0.242 |  |  |  |  |  |  |  |  |  |  |  |  |
| 73 | W | 0.276 | 0.228 | 0.323 |  |  |  |  |  |  |  |  |  |  |  |  |
| 74 | M |  |  |  | 0.226 | 0.188 | 0.263 |  |  |  |  |  |  |  |  |  |
| 74 | W |  |  |  | 0.346 | 0.305 | 0.386 |  |  |  |  |  |  |  |  |  |
| 75 | M | 0.226 | 0.168 | 0.285 |  |  |  | 0.246 | 0.195 | 0.298 |  |  |  |  |  |  |
| 75 | W | 0.302 | 0.246 | 0.358 |  |  |  | 0.405 | 0.349 | 0.460 |  |  |  |  |  |  |
| 76 | M |  |  |  | 0.258 | 0.216 | 0.300 |  |  |  |  |  |  |  |  |  |
| 76 | W |  |  |  | 0.369 | 0.329 | 0.409 |  |  |  |  |  |  |  |  |  |
| 77 | M |  |  |  |  |  |  |  |  |  |  |  |  |  |  |  |
| 77 | W |  |  |  |  |  |  |  |  |  |  |  |  |  |  |  |
| 78 | M |  |  |  | 0.302 | 0.255 | 0.349 |  |  |  |  |  |  |  |  |  |
| 78 | W |  |  |  | 0.404 | 0.363 | 0.445 |  |  |  |  |  |  |  |  |  |
| 79 | M |  |  |  |  |  |  | 0.319 | 0.269 | 0.368 |  |  |  |  |  |  |
| 79 | W |  |  |  |  |  |  | 0.457 | 0.408 | 0.506 |  |  |  |  |  |  |
| 80 | M |  |  |  | 0.356 | 0.298 | 0.415 |  |  |  | 0.312 | 0.238 | 0.387 |  |  |  |
| 80 | W |  |  |  | 0.448 | 0.397 | 0.499 |  |  |  | 0.485 | 0.413 | 0.557 |  |  |  |
| 81 | M |  |  |  |  |  |  | 0.375 | 0.321 | 0.428 |  |  |  |  |  |  |
| 81 | W |  |  |  |  |  |  | 0.501 | 0.452 | 0.550 |  |  |  |  |  |  |
| 82 | M |  |  |  |  |  |  |  |  |  |  |  |  |  |  |  |
| 82 | W |  |  |  |  |  |  |  |  |  |  |  |  |  |  |  |
| 83 | M |  |  |  |  |  |  | 0.442 | 0.383 | 0.500 |  |  |  |  |  |  |
| 83 | W |  |  |  |  |  |  | 0.553 | 0.503 | 0.603 |  |  |  |  |  |  |
| 84 | M |  |  |  |  |  |  |  |  |  | 0.436 | 0.365 | 0.507 | 0.396 | 0.265 | 0.528 |
| 84 | W |  |  |  |  |  |  |  |  |  | 0.582 | 0.522 | 0.642 |  |  |  |
| 85 | M |  |  |  |  |  |  | 0.516 | 0.448 | 0.584 |  |  |  |  |  |  |
| 85 | W |  |  |  |  |  |  | 0.611 | 0.554 | 0.669 |  |  |  | 0.577 | 0.458 | 0.697 |
| 86 | M |  |  |  |  |  |  |  |  |  | 0.515 | 0.443 | 0.588 |  |  |  |
| 86 | W |  |  |  |  |  |  |  |  |  | 0.642 | 0.584 | 0.700 |  |  |  |
| 87 | M |  |  |  |  |  |  |  |  |  |  |  |  |  |  |  |
| 87 | W |  |  |  |  |  |  |  |  |  |  |  |  |  |  |  |
| 88 | M |  |  |  |  |  |  |  |  |  | 0.599 | 0.525 | 0.673 | 0.570 | 0.451 | 0.689 |
| 88 | W |  |  |  |  |  |  |  |  |  | 0.704 | 0.645 | 0.762 |  |  |  |
| 89 | M |  |  |  |  |  |  |  |  |  |  |  |  |  |  |  |
| 89 | W |  |  |  |  |  |  |  |  |  |  |  |  | 0.708 | 0.616 | 0.801 |
| 90 | M |  |  |  |  |  |  |  |  |  | 0.681 | 0.604 | 0.759 | 0.663 | 0.553 | 0.773 |
| 90 | W |  |  |  |  |  |  |  |  |  | 0.763 | 0.702 | 0.825 | 0.773 | 0.692 | 0.854 |
| 91 | M |  |  |  |  |  |  |  |  |  |  |  |  |  |  |  |
| 91 | W |  |  |  |  |  |  |  |  |  |  |  |  |  |  |  |
| 92 | M |  |  |  |  |  |  |  |  |  |  |  |  | 0.749 | 0.651 | 0.847 |
| 92 | W |  |  |  |  |  |  |  |  |  |  |  |  | 0.830 | 0.759 | 0.901 |
| 93 | M |  |  |  |  |  |  |  |  |  |  |  |  |  |  |  |
| 93 | W |  |  |  |  |  |  |  |  |  |  |  |  |  |  |  |
| 94 | M |  |  |  |  |  |  |  |  |  |  |  |  | 0.822 | 0.736 | 0.908 |
| 94 | W |  |  |  |  |  |  |  |  |  |  |  |  |  |  |  |
| 95 | M |  |  |  |  |  |  |  |  |  |  |  |  |  |  |  |
| 95 | W |  |  |  |  |  |  |  |  |  |  |  |  | 0.879 | 0.816 | 0.941 |

**Supplementary table 16.** Predicted probabilities of **IADL** limitations by age and sex adjusted for income and education (**model 2**). **Northern Europe**, data for figure 2. (PP, predicted probability. LCI, lower confidence interval. UCI, upper confidence interval).

|  |  | Cohort 1940-1944 | | | Cohort 1935-1939 | | | Cohort 1930-1934 | | | Cohort 1925-1929 | | | Cohort 1920-1924 | | |
| --- | --- | --- | --- | --- | --- | --- | --- | --- | --- | --- | --- | --- | --- | --- | --- | --- |
| Age | Sex | PP | LCI | UCI | PP | LCI | UCI | PP | LCI | UCI | PP | LCI | UCI | PP | LCI | UCI |
| 62 | M | 0.057 | 0.040 | 0.073 |  |  |  |  |  |  |  |  |  |  |  |  |
| 62 | W | 0.107 | 0.083 | 0.131 |  |  |  |  |  |  |  |  |  |  |  |  |
| … |  |  |  |  |  |  |  |  |  |  |  |  |  |  |  |  |
| 65 | M | 0.059 | 0.044 | 0.074 |  |  |  |  |  |  |  |  |  |  |  |  |
| 65 | W | 0.103 | 0.083 | 0.123 |  |  |  |  |  |  |  |  |  |  |  |  |
| 66 | M |  |  |  |  |  |  |  |  |  |  |  |  |  |  |  |
| 66 | W |  |  |  |  |  |  |  |  |  |  |  |  |  |  |  |
| 67 | M |  |  |  | 0.070 | 0.055 | 0.085 |  |  |  |  |  |  |  |  |  |
| 67 | W |  |  |  | 0.126 | 0.106 | 0.146 |  |  |  |  |  |  |  |  |  |
| 68 | M |  |  |  |  |  |  |  |  |  |  |  |  |  |  |  |
| 68 | W |  |  |  |  |  |  |  |  |  |  |  |  |  |  |  |
| 69 | M | 0.079 | 0.062 | 0.096 |  |  |  |  |  |  |  |  |  |  |  |  |
| 69 | W | 0.115 | 0.094 | 0.136 |  |  |  |  |  |  |  |  |  |  |  |  |
| 70 | M |  |  |  | 0.080 | 0.067 | 0.094 |  |  |  |  |  |  |  |  |  |
| 70 | W |  |  |  | 0.132 | 0.115 | 0.149 |  |  |  |  |  |  |  |  |  |
| 71 | M | 0.097 | 0.078 | 0.117 |  |  |  |  |  |  |  |  |  |  |  |  |
| 71 | W | 0.130 | 0.108 | 0.152 |  |  |  |  |  |  |  |  |  |  |  |  |
| 72 | M |  |  |  |  |  |  | 0.099 | 0.079 | 0.120 |  |  |  |  |  |  |
| 72 | W |  |  |  |  |  |  | 0.166 | 0.141 | 0.191 |  |  |  |  |  |  |
| 73 | M | 0.121 | 0.098 | 0.145 |  |  |  |  |  |  |  |  |  |  |  |  |
| 73 | W | 0.150 | 0.125 | 0.175 |  |  |  |  |  |  |  |  |  |  |  |  |
| 74 | M |  |  |  | 0.123 | 0.106 | 0.140 |  |  |  |  |  |  |  |  |  |
| 74 | W |  |  |  | 0.168 | 0.149 | 0.187 |  |  |  |  |  |  |  |  |  |
| 75 | M | 0.151 | 0.121 | 0.180 |  |  |  | 0.123 | 0.103 | 0.143 |  |  |  |  |  |  |
| 75 | W | 0.175 | 0.145 | 0.206 |  |  |  | 0.187 | 0.165 | 0.210 |  |  |  |  |  |  |
| 76 | M |  |  |  | 0.156 | 0.135 | 0.176 |  |  |  |  |  |  |  |  |  |
| 76 | W |  |  |  | 0.197 | 0.176 | 0.218 |  |  |  |  |  |  |  |  |  |
| 77 | M |  |  |  |  |  |  |  |  |  | 0.156 | 0.125 | 0.187 |  |  |  |
| 77 | W |  |  |  |  |  |  |  |  |  | 0.238 | 0.206 | 0.271 |  |  |  |
| 78 | M |  |  |  | 0.197 | 0.172 | 0.222 |  |  |  |  |  |  |  |  |  |
| 78 | W |  |  |  | 0.232 | 0.208 | 0.257 |  |  |  |  |  |  |  |  |  |
| 79 | M |  |  |  |  |  |  | 0.201 | 0.176 | 0.227 |  |  |  |  |  |  |
| 79 | W |  |  |  |  |  |  | 0.258 | 0.232 | 0.284 |  |  |  |  |  |  |
| 80 | M |  |  |  | 0.245 | 0.212 | 0.277 |  |  |  | 0.201 | 0.170 | 0.231 |  |  |  |
| 80 | W |  |  |  | 0.273 | 0.242 | 0.303 |  |  |  | 0.281 | 0.251 | 0.310 |  |  |  |
| 81 | M |  |  |  |  |  |  | 0.256 | 0.227 | 0.285 |  |  |  |  |  |  |
| 81 | W |  |  |  |  |  |  | 0.305 | 0.278 | 0.333 |  |  |  |  |  |  |
| 82 | M |  |  |  |  |  |  |  |  |  |  |  |  | 0.255 | 0.197 | 0.314 |
| 82 | W |  |  |  |  |  |  |  |  |  |  |  |  | 0.354 | 0.295 | 0.414 |
| 83 | M |  |  |  |  |  |  | 0.318 | 0.285 | 0.351 |  |  |  |  |  |  |
| 83 | W |  |  |  |  |  |  | 0.357 | 0.327 | 0.388 |  |  |  |  |  |  |
| 84 | M |  |  |  |  |  |  |  |  |  | 0.331 | 0.294 | 0.367 |  |  |  |
| 84 | W |  |  |  |  |  |  |  |  |  | 0.396 | 0.362 | 0.430 |  |  |  |
| 85 | M |  |  |  |  |  |  | 0.383 | 0.342 | 0.424 |  |  |  | 0.331 | 0.271 | 0.391 |
| 85 | W |  |  |  |  |  |  | 0.412 | 0.374 | 0.450 |  |  |  | 0.424 | 0.367 | 0.482 |
| 86 | M |  |  |  |  |  |  |  |  |  | 0.408 | 0.368 | 0.448 |  |  |  |
| 86 | W |  |  |  |  |  |  |  |  |  | 0.461 | 0.425 | 0.498 |  |  |  |
| 87 | M |  |  |  |  |  |  |  |  |  |  |  |  |  |  |  |
| 87 | W |  |  |  |  |  |  |  |  |  |  |  |  |  |  |  |
| 88 | M |  |  |  |  |  |  |  |  |  | 0.488 | 0.443 | 0.533 |  |  |  |
| 88 | W |  |  |  |  |  |  |  |  |  | 0.528 | 0.487 | 0.568 |  |  |  |
| 89 | M |  |  |  |  |  |  |  |  |  |  |  |  | 0.514 | 0.452 | 0.577 |
| 89 | W |  |  |  |  |  |  |  |  |  |  |  |  | 0.578 | 0.522 | 0.634 |
| 90 | M |  |  |  |  |  |  |  |  |  | 0.566 | 0.515 | 0.617 |  |  |  |
| 90 | W |  |  |  |  |  |  |  |  |  | 0.591 | 0.545 | 0.637 |  |  |  |
| 91 | M |  |  |  |  |  |  |  |  |  |  |  |  | 0.604 | 0.543 | 0.666 |
| 91 | W |  |  |  |  |  |  |  |  |  |  |  |  | 0.650 | 0.595 | 0.704 |
| 92 | M |  |  |  |  |  |  |  |  |  |  |  |  |  |  |  |
| 92 | W |  |  |  |  |  |  |  |  |  |  |  |  |  |  |  |
| 93 | M |  |  |  |  |  |  |  |  |  |  |  |  | 0.684 | 0.625 | 0.743 |
| 93 | W |  |  |  |  |  |  |  |  |  |  |  |  | 0.713 | 0.660 | 0.767 |
| 94 | M |  |  |  |  |  |  |  |  |  |  |  |  |  |  |  |
| 94 | W |  |  |  |  |  |  |  |  |  |  |  |  | 0.768 | 0.715 | 0.822 |
| 95 | M |  |  |  |  |  |  |  |  |  |  |  |  | 0.752 | 0.693 | 0.811 |

**Supplementary table 17.** Predicted probabilities of **IADL** limitations by age and sex adjusted for income and education (**model 2**). **Southern Europe**, data for figure 2. (PP, predicted probability. LCI, lower confidence interval. UCI, upper confidence interval).

|  |  | Cohort 1940-1944 | | | Cohort 1935-1939 | | | Cohort 1930-1934 | | | Cohort 1925-1929 | | | Cohort 1920-1924 | | |
| --- | --- | --- | --- | --- | --- | --- | --- | --- | --- | --- | --- | --- | --- | --- | --- | --- |
| Age | Sex | PP | LCI | UCI | PP | LCI | UCI | PP | LCI | UCI | PP | LCI | UCI | PP | LCI | UCI |
| 62 | M | 0.092 | 0.070 | 0.114 |  |  |  |  |  |  |  |  |  |  |  |  |
| 62 | W | 0.171 | 0.145 | 0.197 |  |  |  |  |  |  |  |  |  |  |  |  |
| … |  |  |  |  |  |  |  |  |  |  |  |  |  |  |  |  |
| 65 | M | 0.091 | 0.072 | 0.109 |  |  |  |  |  |  |  |  |  |  |  |  |
| 65 | W | 0.170 | 0.149 | 0.191 |  |  |  |  |  |  |  |  |  |  |  |  |
| 66 | M |  |  |  |  |  |  |  |  |  |  |  |  |  |  |  |
| 66 | W |  |  |  |  |  |  |  |  |  |  |  |  |  |  |  |
| 67 | M |  |  |  | 0.111 | 0.094 | 0.128 |  |  |  |  |  |  |  |  |  |
| 67 | W |  |  |  | 0.203 | 0.182 | 0.223 |  |  |  |  |  |  |  |  |  |
| 68 | M |  |  |  |  |  |  |  |  |  |  |  |  |  |  |  |
| 68 | W |  |  |  |  |  |  |  |  |  |  |  |  |  |  |  |
| 69 | M | 0.107 | 0.088 | 0.125 |  |  |  |  |  |  |  |  |  |  |  |  |
| 69 | W | 0.194 | 0.172 | 0.217 |  |  |  |  |  |  |  |  |  |  |  |  |
| 70 | M |  |  |  | 0.120 | 0.105 | 0.135 |  |  |  |  |  |  |  |  |  |
| 70 | W |  |  |  | 0.218 | 0.200 | 0.236 |  |  |  |  |  |  |  |  |  |
| 71 | M | 0.124 | 0.104 | 0.144 |  |  |  |  |  |  |  |  |  |  |  |  |
| 71 | W | 0.219 | 0.195 | 0.243 |  |  |  |  |  |  |  |  |  |  |  |  |
| 72 | M |  |  |  |  |  |  | 0.146 | 0.126 | 0.166 |  |  |  |  |  |  |
| 72 | W |  |  |  |  |  |  | 0.257 | 0.231 | 0.283 |  |  |  |  |  |  |
| 73 | M | 0.148 | 0.125 | 0.171 |  |  |  |  |  |  |  |  |  |  |  |  |
| 73 | W | 0.250 | 0.223 | 0.277 |  |  |  |  |  |  |  |  |  |  |  |  |
| 74 | M |  |  |  | 0.162 | 0.145 | 0.179 |  |  |  |  |  |  |  |  |  |
| 74 | W |  |  |  | 0.277 | 0.256 | 0.298 |  |  |  |  |  |  |  |  |  |
| 75 | M | 0.179 | 0.151 | 0.207 |  |  |  | 0.170 | 0.152 | 0.189 |  |  |  |  |  |  |
| 75 | W | 0.289 | 0.256 | 0.322 |  |  |  | 0.292 | 0.268 | 0.316 |  |  |  |  |  |  |
| 76 | M |  |  |  | 0.196 | 0.178 | 0.215 |  |  |  |  |  |  |  |  |  |
| 76 | W |  |  |  | 0.320 | 0.298 | 0.342 |  |  |  |  |  |  |  |  |  |
| 77 | M |  |  |  |  |  |  |  |  |  | 0.205 | 0.175 | 0.234 |  |  |  |
| 77 | W |  |  |  |  |  |  |  |  |  | 0.339 | 0.305 | 0.374 |  |  |  |
| 78 | M |  |  |  | 0.239 | 0.218 | 0.260 |  |  |  |  |  |  |  |  |  |
| 78 | W |  |  |  | 0.370 | 0.346 | 0.394 |  |  |  |  |  |  |  |  |  |
| 79 | M |  |  |  |  |  |  | 0.252 | 0.229 | 0.275 |  |  |  |  |  |  |
| 79 | W |  |  |  |  |  |  | 0.395 | 0.367 | 0.423 |  |  |  |  |  |  |
| 80 | M |  |  |  | 0.290 | 0.262 | 0.317 |  |  |  | 0.251 | 0.223 | 0.280 |  |  |  |
| 80 | W |  |  |  | 0.426 | 0.396 | 0.456 |  |  |  | 0.400 | 0.368 | 0.431 |  |  |  |
| 81 | M |  |  |  |  |  |  | 0.308 | 0.283 | 0.333 |  |  |  |  |  |  |
| 81 | W |  |  |  |  |  |  | 0.458 | 0.430 | 0.486 |  |  |  |  |  |  |
| 82 | M |  |  |  |  |  |  |  |  |  |  |  |  | 0.296 | 0.239 | 0.353 |
| 82 | W |  |  |  |  |  |  |  |  |  |  |  |  | 0.454 | 0.395 | 0.514 |
| 83 | M |  |  |  |  |  |  | 0.373 | 0.345 | 0.401 |  |  |  |  |  |  |
| 83 | W |  |  |  |  |  |  | 0.524 | 0.496 | 0.553 |  |  |  |  |  |  |
| 84 | M |  |  |  |  |  |  |  |  |  | 0.383 | 0.349 | 0.418 |  |  |  |
| 84 | W |  |  |  |  |  |  |  |  |  | 0.545 | 0.512 | 0.579 |  |  |  |
| 85 | M |  |  |  |  |  |  | 0.443 | 0.408 | 0.478 |  |  |  | 0.372 | 0.314 | 0.429 |
| 85 | W |  |  |  |  |  |  | 0.591 | 0.557 | 0.625 |  |  |  | 0.538 | 0.483 | 0.593 |
| 86 | M |  |  |  |  |  |  |  |  |  | 0.463 | 0.425 | 0.500 |  |  |  |
| 86 | W |  |  |  |  |  |  |  |  |  | 0.621 | 0.587 | 0.654 |  |  |  |
| 87 | M |  |  |  |  |  |  |  |  |  |  |  |  |  |  |  |
| 87 | W |  |  |  |  |  |  |  |  |  |  |  |  |  |  |  |
| 88 | M |  |  |  |  |  |  |  |  |  | 0.544 | 0.504 | 0.584 |  |  |  |
| 88 | W |  |  |  |  |  |  |  |  |  | 0.692 | 0.658 | 0.725 |  |  |  |
| 89 | M |  |  |  |  |  |  |  |  |  |  |  |  | 0.552 | 0.493 | 0.611 |
| 89 | W |  |  |  |  |  |  |  |  |  |  |  |  | 0.707 | 0.659 | 0.754 |
| 90 | M |  |  |  |  |  |  |  |  |  | 0.623 | 0.578 | 0.668 |  |  |  |
| 90 | W |  |  |  |  |  |  |  |  |  | 0.755 | 0.720 | 0.791 | 0.778 | 0.736 | 0.821 |
| 91 | M |  |  |  |  |  |  |  |  |  |  |  |  | 0.642 | 0.585 | 0.699 |
| 91 | W |  |  |  |  |  |  |  |  |  |  |  |  |  |  |  |
| 92 | M |  |  |  |  |  |  |  |  |  |  |  |  | 0.723 | 0.669 | 0.777 |
| 92 | W |  |  |  |  |  |  |  |  |  |  |  |  |  |  |  |
| 93 | M |  |  |  |  |  |  |  |  |  |  |  |  |  |  |  |
| 93 | W |  |  |  |  |  |  |  |  |  |  |  |  | 0.837 | 0.800 | 0.874 |
| 94 | M |  |  |  |  |  |  |  |  |  |  |  |  | 0.792 | 0.741 | 0.843 |
| 94 | W |  |  |  |  |  |  |  |  |  |  |  |  | 0.883 | 0.850 | 0.916 |

**Supplementary table 18.** Predicted probabilities of **IADL** limitations by age and sex adjusted for income and education (**model 2**). **Western Europe**, data for figure 2. (PP, predicted probability. LCI, lower confidence interval. UCI, upper confidence interval).

|  |  | Cohort 1940-1944 | | | Cohort 1935-1939 | | | Cohort 1930-1934 | | | Cohort 1925-1929 | | | Cohort 1920-1924 | | |
| --- | --- | --- | --- | --- | --- | --- | --- | --- | --- | --- | --- | --- | --- | --- | --- | --- |
| Age | Sex | PP | LCI | UCI | PP | LCI | UCI | PP | LCI | UCI | PP | LCI | UCI | PP | LCI | UCI |
| 62 | M | 0.075 | 0.058 | 0.093 |  |  |  |  |  |  |  |  |  |  |  |  |
| 62 | W | 0.112 | 0.094 | 0.130 |  |  |  |  |  |  |  |  |  |  |  |  |
| … |  |  |  |  |  |  |  |  |  |  |  |  |  |  |  |  |
| 65 | M | 0.083 | 0.067 | 0.099 |  |  |  |  |  |  |  |  |  |  |  |  |
| 65 | W | 0.121 | 0.104 | 0.138 |  |  |  |  |  |  |  |  |  |  |  |  |
| 66 | M |  |  |  |  |  |  |  |  |  |  |  |  |  |  |  |
| 66 | W |  |  |  |  |  |  |  |  |  |  |  |  |  |  |  |
| 67 | M |  |  |  | 0.086 | 0.073 | 0.100 |  |  |  |  |  |  |  |  |  |
| 67 | W |  |  |  | 0.127 | 0.112 | 0.141 |  |  |  |  |  |  |  |  |  |
| 68 | M |  |  |  |  |  |  |  |  |  |  |  |  |  |  |  |
| 68 | W |  |  |  |  |  |  |  |  |  |  |  |  |  |  |  |
| 69 | M | 0.108 | 0.089 | 0.126 |  |  |  |  |  |  |  |  |  |  |  |  |
| 69 | W | 0.149 | 0.130 | 0.169 |  |  |  |  |  |  |  |  |  |  |  |  |
| 70 | M |  |  |  | 0.104 | 0.091 | 0.117 |  |  |  |  |  |  |  |  |  |
| 70 | W |  |  |  | 0.149 | 0.135 | 0.164 |  |  |  |  |  |  |  |  |  |
| 71 | M | 0.125 | 0.105 | 0.144 |  |  |  |  |  |  |  |  |  |  |  |  |
| 71 | W | 0.168 | 0.147 | 0.189 |  |  |  |  |  |  |  |  |  |  |  |  |
| 72 | M |  |  |  |  |  |  | 0.113 | 0.098 | 0.129 |  |  |  |  |  |  |
| 72 | W |  |  |  |  |  |  | 0.163 | 0.144 | 0.182 |  |  |  |  |  |  |
| 73 | M | 0.144 | 0.121 | 0.167 |  |  |  |  |  |  |  |  |  |  |  |  |
| 73 | W | 0.189 | 0.165 | 0.214 |  |  |  |  |  |  |  |  |  |  |  |  |
| 74 | M |  |  |  | 0.156 | 0.139 | 0.172 |  |  |  |  |  |  |  |  |  |
| 74 | W |  |  |  | 0.209 | 0.191 | 0.227 |  |  |  |  |  |  |  |  |  |
| 75 | M | 0.166 | 0.138 | 0.194 |  |  |  | 0.148 | 0.131 | 0.164 |  |  |  |  |  |  |
| 75 | W | 0.213 | 0.183 | 0.243 |  |  |  | 0.205 | 0.185 | 0.224 |  |  |  |  |  |  |
| 76 | M |  |  |  | 0.189 | 0.170 | 0.207 |  |  |  |  |  |  |  |  |  |
| 76 | W |  |  |  | 0.246 | 0.226 | 0.265 |  |  |  |  |  |  |  |  |  |
| 77 | M |  |  |  |  |  |  |  |  |  | 0.167 | 0.144 | 0.190 |  |  |  |
| 77 | W |  |  |  |  |  |  |  |  |  | 0.231 | 0.207 | 0.256 |  |  |  |
| 78 | M |  |  |  | 0.226 | 0.204 | 0.248 |  |  |  |  |  |  |  |  |  |
| 78 | W |  |  |  | 0.285 | 0.262 | 0.307 |  |  |  |  |  |  |  |  |  |
| 79 | M |  |  |  |  |  |  | 0.241 | 0.219 | 0.263 |  |  |  |  |  |  |
| 79 | W |  |  |  |  |  |  | 0.309 | 0.285 | 0.334 |  |  |  |  |  |  |
| 80 | M |  |  |  | 0.265 | 0.236 | 0.294 |  |  |  | 0.227 | 0.202 | 0.251 |  |  |  |
| 80 | W |  |  |  | 0.325 | 0.296 | 0.354 |  |  |  | 0.300 | 0.275 | 0.324 |  |  |  |
| 81 | M |  |  |  |  |  |  | 0.297 | 0.272 | 0.322 |  |  |  |  |  |  |
| 81 | W |  |  |  |  |  |  | 0.368 | 0.342 | 0.394 |  |  |  |  |  |  |
| 82 | M |  |  |  |  |  |  |  |  |  |  |  |  | 0.262 | 0.216 | 0.308 |
| 82 | W |  |  |  |  |  |  |  |  |  |  |  |  | 0.343 | 0.299 | 0.387 |
| 83 | M |  |  |  |  |  |  | 0.356 | 0.326 | 0.385 |  |  |  |  |  |  |
| 83 | W |  |  |  |  |  |  | 0.427 | 0.399 | 0.456 |  |  |  |  |  |  |
| 84 | M |  |  |  |  |  |  |  |  |  | 0.376 | 0.344 | 0.408 |  |  |  |
| 84 | W |  |  |  |  |  |  |  |  |  | 0.458 | 0.428 | 0.488 |  |  |  |
| 85 | M |  |  |  |  |  |  | 0.414 | 0.377 | 0.452 |  |  |  | 0.356 | 0.307 | 0.406 |
| 85 | W |  |  |  |  |  |  | 0.484 | 0.449 | 0.520 |  |  |  | 0.445 | 0.401 | 0.489 |
| 86 | M |  |  |  |  |  |  |  |  |  | 0.457 | 0.421 | 0.493 |  |  |  |
| 86 | W |  |  |  |  |  |  |  |  |  | 0.537 | 0.505 | 0.569 |  |  |  |
| 87 | M |  |  |  |  |  |  |  |  |  |  |  |  |  |  |  |
| 87 | W |  |  |  |  |  |  |  |  |  |  |  |  |  |  |  |
| 88 | M |  |  |  |  |  |  |  |  |  | 0.535 | 0.495 | 0.575 |  |  |  |
| 88 | W |  |  |  |  |  |  |  |  |  | 0.609 | 0.574 | 0.644 |  |  |  |
| 89 | M |  |  |  |  |  |  |  |  |  | 0.605 | 0.559 | 0.652 | 0.562 | 0.509 | 0.615 |
| 89 | W |  |  |  |  |  |  |  |  |  |  |  |  | 0.644 | 0.600 | 0.688 |
| 90 | M |  |  |  |  |  |  |  |  |  |  |  |  | 0.654 | 0.602 | 0.706 |
| 90 | W |  |  |  |  |  |  |  |  |  | 0.672 | 0.632 | 0.712 | 0.725 | 0.683 | 0.767 |
| 91 | M |  |  |  |  |  |  |  |  |  |  |  |  |  |  |  |
| 91 | W |  |  |  |  |  |  |  |  |  |  |  |  |  |  |  |
| 92 | M |  |  |  |  |  |  |  |  |  |  |  |  | 0.731 | 0.680 | 0.782 |
| 92 | W |  |  |  |  |  |  |  |  |  |  |  |  | 0.790 | 0.750 | 0.831 |
| 93 | M |  |  |  |  |  |  |  |  |  |  |  |  |  |  |  |
| 93 | W |  |  |  |  |  |  |  |  |  |  |  |  |  |  |  |
| 94 | M |  |  |  |  |  |  |  |  |  |  |  |  | 0.793 | 0.743 | 0.843 |
| 94 | W |  |  |  |  |  |  |  |  |  |  |  |  | 0.840 | 0.801 | 0.880 |
